# Structural and Functional Studies of Nonstructural Protein 2 of the Hepatitis C Virus Reveal Its Key Role as Organizer of Virion Assembly

**DOI:** 10.1371/journal.ppat.1001233

**Published:** 2010-12-16

**Authors:** Vlastimil Jirasko, Roland Montserret, Ji Young Lee, Jérôme Gouttenoire, Darius Moradpour, Francois Penin, Ralf Bartenschlager

**Affiliations:** 1 Department of Infectious Diseases, Molecular Virology, University of Heidelberg, Heidelberg, Germany; 2 Institut de Biologie et Chimie des Protéines, UMR 5086 CNRS, Université de Lyon, Lyon, France; 3 Division of Gastroenterology and Hepatology, Centre Hospitalier Universitaire Vaudois, University of Lausanne, Lausanne, Switzerland; Washington University School of Medicine, United States of America

## Abstract

Non-structural protein 2 (NS2) plays an important role in hepatitis C virus (HCV) assembly, but neither the exact contribution of this protein to the assembly process nor its complete structure are known. In this study we used a combination of genetic, biochemical and structural methods to decipher the role of NS2 in infectious virus particle formation. A large panel of NS2 mutations targeting the N-terminal membrane binding region was generated. They were selected based on a membrane topology model that we established by determining the NMR structures of N-terminal NS2 transmembrane segments. Mutants affected in virion assembly, but not RNA replication, were selected for pseudoreversion in cell culture. Rescue mutations restoring virus assembly to various degrees emerged in E2, p7, NS3 and NS2 itself arguing for an interaction between these proteins. To confirm this assumption we developed a fully functional JFH1 genome expressing an N-terminally tagged NS2 demonstrating efficient pull-down of NS2 with p7, E2 and NS3 and, to a lower extent, NS5A. Several of the mutations blocking virus assembly disrupted some of these interactions that were restored to various degrees by those pseudoreversions that also restored assembly. Immunofluorescence analyses revealed a time-dependent NS2 colocalization with E2 at sites close to lipid droplets (LDs) together with NS3 and NS5A. Importantly, NS2 of a mutant defective in assembly abrogates NS2 colocalization around LDs with E2 and NS3, which is restored by a pseudoreversion in p7, whereas NS5A is recruited to LDs in an NS2-independent manner. In conclusion, our results suggest that NS2 orchestrates HCV particle formation by participation in multiple protein-protein interactions required for their recruitment to assembly sites in close proximity of LDs.

## Author Summary

Formation of infectious virus particles (assembly) is a complex process by which structural proteins and the viral genome must be transferred to the same subcellular sites to allow their direct or indirect interaction. In case of the hepatitis C virus (HCV), this process appears to take place in close proximity of lipid droplets (LDs) and requires in addition to the structural proteins core, envelope glycoprotein 1 (E1) and E2 two auxiliary factors, designated p7 and nonstructural protein 2 (NS2), contributing to virion formation by unknown mechanisms. In this study we used a combination of structural, genetic and biochemical assays to study the role of NS2 in HCV assembly. By using nuclear magnetic resonance spectroscopy of NS2 peptides we established a membrane topology model of the amino-terminal membrane binding domain of NS2. We found that this protein participates in multiple interactions with E2, p7, NS3 and NS5A that appear to recruit the viral proteins to sites in close proximity of LDs. In this respect, NS2 is a key organizer of the assembly of infectious HCV particles.

## Introduction

Chronic infection with the hepatitis C virus (HCV) is amongst the most frequent causes of liver cirrhosis and hepatocellular carcinoma [1]. About 3% of the world population is persistently infected with this virus and inspite of significant decline of new infections, owing to the long incubation period, a profound rise in the frequency of long-term complications such as steatosis, cirrhosis and liver cancer is expected [2].

HCV is the predominant member of the genus Hepacivirus in the family Flaviviridae. These viruses are enveloped and possess a single strand RNA of positive polarity. In case of HCV the genome has a length of ∼9.6 kb and it encodes a single polyprotein that is cleaved co- and post-translationally by cellular and viral proteases into 10 different products [3], [4]: core, envelope protein 1 (E1), E2, p7, nonstructural protein 2 (NS2), NS3, NS4A, NS4B, NS5A and NS5B. Core, E1 and E2 are the main viral constituents of the HCV particle. P7 and NS2 are essential ‘co-factors’ for virus assembly [5], [6], but dispensable for RNA replication [7]. This process is catalyzed by the concerted action of NS3 to NS5B proteins forming –together with cellular proteins- a membrane-associated replicase complex [8].

Studies of HCV assembly and release have become possible with the identification of the genotype 2a isolate JFH1 that efficiently replicates in the human hepatoma cell line Huh-7 and supports production of infectious virus particles [9]. This culture system has been improved by the identification of virus titer-enhancing mutations increasing infectivity yields by up to 1,000-fold [10]–[12] and the construction of JFH1 chimeras in which the region encoding core to NS2 has been replaced by analogous genome fragments from other HCV isolates [13], [14].

With the advent of these cell culture systems, first insights into HCV assembly and the roles of p7 and NS2 in this process could be gained. P7 is a small hydrophobic protein composed of two transmembrane segments (TMS) [15], [16]. It is capable to form hexa- or heptameric complexes that can act as a viroporin [17]–[19]. P7 is dispensable for RNA replication [7], but crucial for infectivity in vivo [20] likely because of its critical role in virus particle assembly [5], [21]. Whether p7 is a component of the virion is discussed controversially [21], [22].

NS2 is a 217 amino acids (aa) long cysteine-protease composed of a highly hydrophobic N-terminal membrane binding domain (MBD) and a C-terminal globular and cytosolic protease subdomain. The latter is capable to form dimers creating a composite active site [23]. This protease is not directly required for RNA replication, but has to be cleaved off the N-terminus of NS3 to allow formation of an active replicase [24]. Recently it was shown that NS2 is essential for HCV assembly [5], [6]. Interestingly, protease activity is not required for particle formation, but rather global integrity of both NS2 subdomains [25], [26]. Although the precise mode-of-action of NS2 during HCV assembly is not known, a recent study suggests that this protein acts at a late stage of infectious particle formation [27].

The exact membrane topology and architecture of the N-terminal MBD of NS2 is not known. It might be composed of three trans-membrane segments (TMS) [28], but alternative models are possible. We have recently shown that TMS1 (aa 1–23) adopts an overall helical fold interrupted by flexible glycine residues at position 10 and 11 [6]. While TMS-1 is clearly predicted as a single membrane-spanning trans-membrane helix, this is less clear for TMS2 and TMS3 that may span the membrane bilayer or reside on its cytosolic surface in a helix-loop-helix configuration.

By using combinations of reverse genetic and biochemical approaches, convincing evidence has been obtained that also factors of the viral replicase are essential for particle formation, most notably NS3 and NS5A [25], [29]–[32]. The latter is a highly phosphorylated RNA binding protein composed of an N-terminal amphipathic alpha-helix serving as a membrane anchor and contributing to targeting of the protein to lipid droplets (LDs) [33], [34], and three domains [35]. Domain I forms a dimer and is essential for RNA replication [36]. Most of domain II is dispensable for replication [30] whereas the C-terminal domain III is essential for virus production, most likely *via* interaction with the core protein [32]. This interaction appears to be regulated by casein kinase II-mediated phosphorylation of NS5A [29].

Assembly of HCV particles is tightly linked to lipid metabolism, LDs and the machinery required for production and secretion of very-low-density lipoproteins (VLDL) [31], [37]–[39]. Several models of HCV assembly have been put forward, but the precise details are unknown (reviewed in [40]). While these models can explain the early steps of nucleocapsid formation, it is unclear how these nucleocapsids acquire the membranous viral envelope and the envelope glycoproteins and how this process is linked to VLDL formation and secretion. NS2 may play a central role in these reactions, but the precise mechanisms are not known [25], [27].

In this study we undertook a detailed structural and functional characterization of the N-terminal MBD of NS2. We solved the NMR-structures of TMS2 and TMS3 and propose a model of NS2 membrane topology. In addition, we performed a structure-activity study of the MBD and established an interaction map of NS2. The data reveal that NS2 serves as a key organizer participating in multiple protein-protein interactions that are required for the assembly of infectious HCV particles.

## Results

### Mapping of transmembrane domains in NS2 and tentative model of its membrane topology

**Figure 1:**
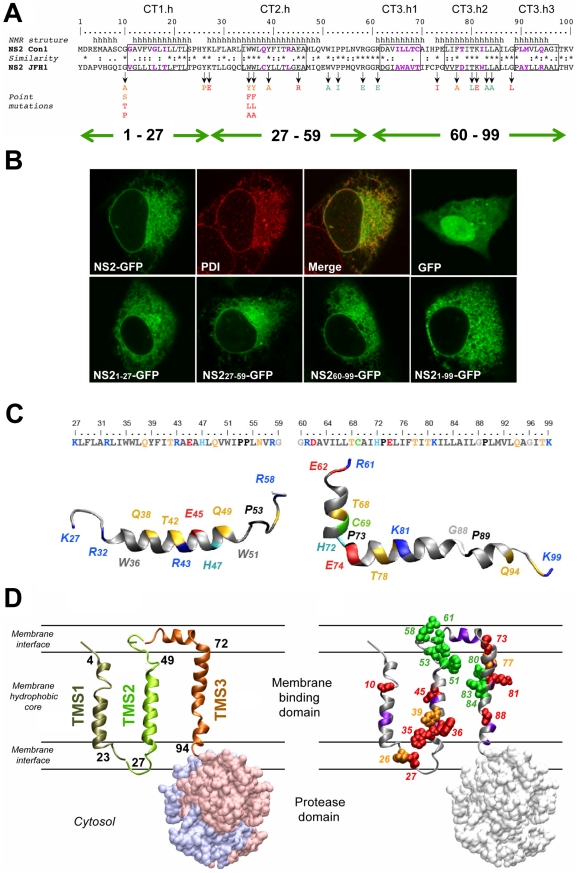
Model of the membrane binding domain of NS2 and its orientation in the membrane. (A) Sequence comparison of the NS2 segment 1–100 from Con1 and JFH1 used to design the NS2 mutants. Amino acids are numbered with respect to NS2. The helical segments in the membrane binding domain deduced from NMR analyses of Con1 NS2 peptides ([6] and this study) are shown at the *top* (h, helical). Identical, highly similar, and similar residues at each position are symbolized by an *asterisk*, a *colon*, and a *dot*, respectively, according to Clustal W convention. Boxes indicate helix segments that were exchanged with the helix-swap mutants (for nomenclature of the mutants see legend to Figure 2). Residues exhibiting strong physico-chemical differences within these boxes are colored *violet*. Arrows indicate residues that were mutated in this study and point to the substituting residues that are color-coded according to their effect on infectious particle assembly: *green*, no significant effect; *orange* and *red*, medium and strong reduction of infectious virus production, respectively. Green double arrows shown in the bottom refer to the three putative TMS as deduced from the predictions. (B) Membrane association mediated by individual NS2 TMS. U-2 OS cells were transiently transfected with pCMVNS2-GFP or pCMVGFP (upper panel) or with pCMVNS2_1-27_-GFP, pCMVNS2_27-59_-GFP, pCMVNS2_60-99_-GFP or pCMVNS2_1-99_-GFP, as indicated in the captions (lower panel). Twenty four to 48 h later cells were fixed and analyzed by confocal laser scanning microscopy. GFP fusion proteins were detected by autofluorescence, ER membrane localization of NS2-GFP was confirmed by indirect immunofluorescence by using PDI as ER marker (upper panel). (C) Ribbon diagrams of representative NMR structures of NS2[27]-[59] and NS2[60-99] (left and right, respectively). Residues are color-coded according to their physicochemical properties. Hydrophobic residues are grey, aromatic residues are dark grey, Gly is light grey, Pro is black. Polar residues are yellow and positively and negatively charged groups of basic and acidic residues are blue and red, respectively. Histidine residues are cyan, the cystein residue is green. Some aa are labelled to provide a visual reference for residue positions in the structures. Figures were generated from structure coordinates (PDB entry: 2KWT and 2KWZ for NS2[27]-[59] and NS2[60-99], respectively) by using the VMD program [67]) and rendered with the POV-Ray software package. (D) Model of NS2 membrane topology. In the left panel TMS 1, 2 and 3 are shown in ribbon representation and colored bronze, green and copper, respectively. The TMS are tentatively positioned in the membrane and the limits of transmembrane helices are given (TMS1, 4–23; TMS2, 27–49; and TMS3, 72–94). The three TMS are represented as separated entities, since their intramolecular and/or intermolecular interactions are not known. The NS2 protease domain is shown in surface representation (side view) with dimer subunits (PDB entry 2HD0) shown in light blue and pink. For simplicity, the MBD for only one protease subunit is shown. The phospholipid bilayer is tentatively and schematically represented. The ribbon model of NS2 MBD in the right panel shows the location of mutated residues analyzed in this study. Side chain atoms of mutated aa are shown as spheres corresponding to van der Waals radius and color-coded as described in panel A to indicate the assembly phenotype. Backbones of residues with low similarity in helix-swap mutants are colored violet.

We reported recently that a transmembrane segment denoted TMS1 was almost invariably predicted in the very N-terminal region (aa 1–23) of NS2, irrespective of the analyzed genotypes and subtypes [6]. TMS in the 23–102 region ([6] and references therein) yielded inconsistent results that depended both on the genotype examined and the method used (data not shown). By using secondary structure predictions and the algorithm developed by Wimley and White to calculate the propensity of an aa sequence to interact with membranes (Figure S1, A and B) we could deduce that the consensus segments 17–45 and 72–96 exhibit a clear propensity to partition into the membrane bilayer and likely include transmembrane helical passages (Figure 1A and supplementary Figure S1). In contrast, the aa segment 49–71 is predicted not to show such properties. Based on these results, the NS2 MBD sequence was divided into the three segments: 1–27, 27–59, and 60–99, each containing a putative transmembrane helix (Figure 1A).

To determine the capacity of these segments to associate with membranes, we analyzed proteins comprising full length NS2 or putative NS2 TMS that were C-terminally fused to green fluorescent protein (GFP) by fluorescence microscopy. The NS2-GFP fusion protein showed a fluorescence pattern that included the nuclear membrane, was strongest in the perinuclear region, and extended in a reticular pattern throughout the cytoplasm (Figure 1B). This pattern corresponds to the endoplasmic reticulum (ER), as corroborated by the colocalization with protein disulfide isomerase (PDI). Each of the predicted TMS of NS2 showed a very similar subcellular localization whereas GFP expressed individually was diffusely distributed throughout the cell including the nucleus. These observations indicate a clear propensity of each of the three segments to associate with membranes.

To gain insight into the structure and membranotropic properties of NS2 segments 27–59 and 60–99, the corresponding peptides of the Con1 strain (1b) designated NS2[27]-[59] and NS2[60-99] were chemically synthesized, purified to homogeneity, and their structures were analyzed by circular dichroism and nuclear magnetic resonance in membrane mimetic environments (for details see supplementary Figure S1 and materials and methods S1). The 3D model structures obtained for both peptides identified one α-helical segment in case of NS2[27]-[59] and three well defined helical segments in case of NS2[60-99] (Figure 1C). Based on physicochemical considerations, a transmembrane orientation of the amphipathic α-helix in TMS2 could only be achieved upon interaction with another complementary TMS neutralizing the polar and charged residues located in the hydrophobic core of the membrane. In case of NS2[60-99], the 35 aa segment including all three helices would be too long for a single transmembrane passage given the average length of transmembrane helices (16 to 25 aa). As the most hydrophobic stretch extends between aa 82–93, and considering that both edges of this stretch include large hydrophobic residues, we assume that segment ∼77–97 forms a TMS. The first small helix (64–69), which includes the short hydrophobic stretch VILL might be located in the membrane interface, possibly in-plane of the membrane.

These considerations together with the available structural data for NS2 TMS1 [6] allow us to propose a model for the membrane association and topology of NS2 MBD (Figure 1D). It would contain three transmembrane, mainly helical segments (TMS1: 4–23; TMS2: 27–49; and TMS3: 72–94), connected by a small cytosolic loop (aa 24–26) and by a luminal segment (aa 50–71) containing a short helix supposed to interact with the membrane interface. Although in this model the three TMS and protease ectodomain are represented as separated entities, given the dimeric structure of the protease domain [23] we expect a packed overall NS2 structure and eventually higher-order complexes of NS2 dimers mediated by intermolecular interactions between MBDs.

### Experimental design to study the NS2 membrane binding domain by reverse genetics

To correlate the structure of NS2 MBD with function, we conducted an extensive mutagenesis of this domain based on the NMR secondary structures in order to identify residues and structural determinants that are most critical for HCV assembly without affecting RNA replication. Mutants with a very low, but still detectable assembly competence were then used to select for pseudoreversions capable to rescue the assembly defect, which was achieved by serial passage of virus in Huh7.5 cells. We anticipated that these pseudoreversions would reside either within NS2, which might be used to refine the structure model, or in other viral proteins that thus would be candidates for interaction with NS2.

**Figure 2:**
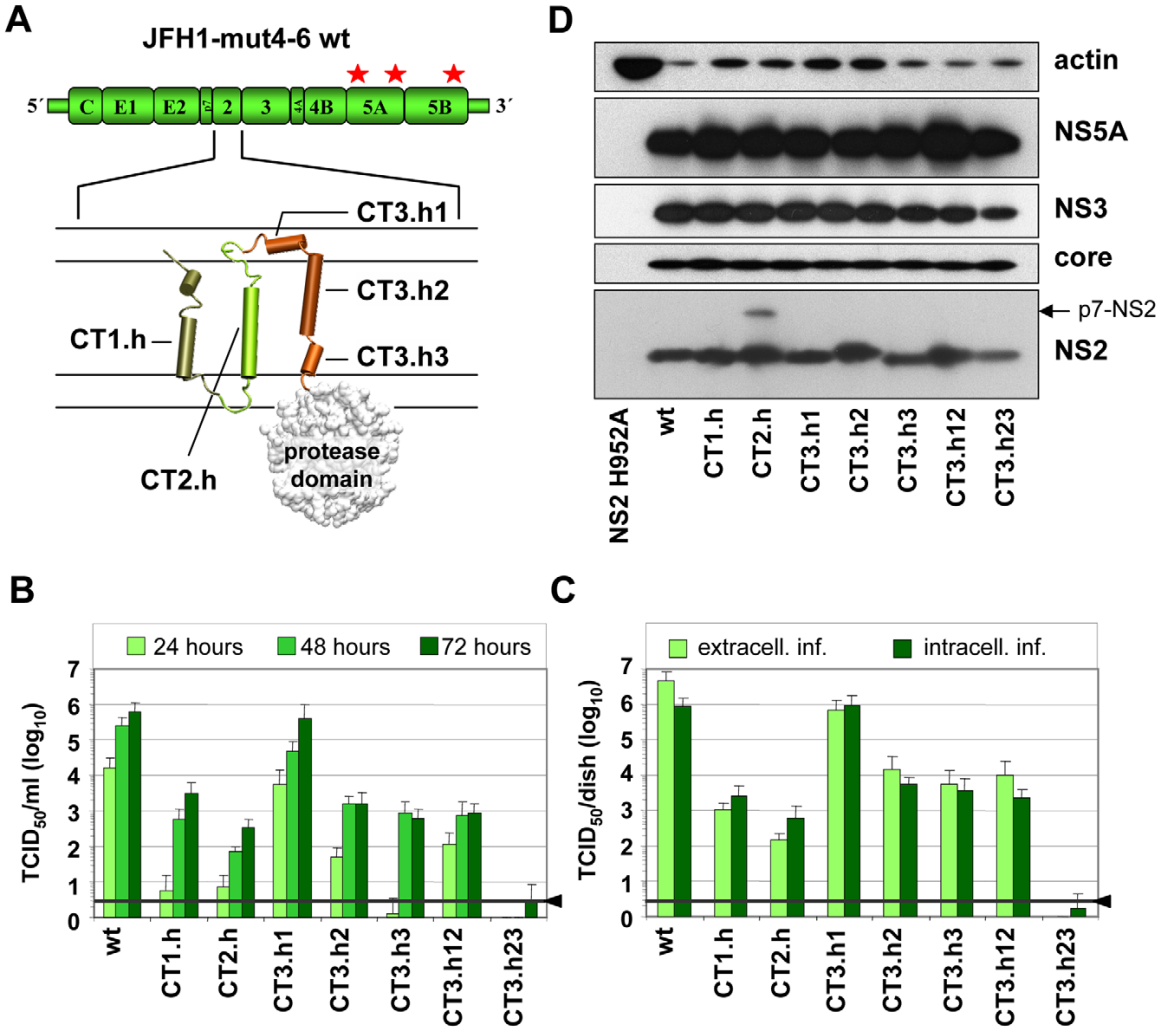
Exchange of helices within the NS2 membrane binding domain and impact on infectious HCV particle production. (A) A schematic diagram of the cell culture adapted JFH1 genome construct (JFH1mut4-6; [10]) used for functional characterization of the NS2 membrane binding domain (MBD) is shown in the top. The titer enhancing mutations are indicated with asterisks. The membrane topology model of the N-terminal MBD is schematically shown below. Helices in individual TMS are represented by cylinders and numbered. Note that TMS3 contains 3 individual helices (h1–h3). C refers to Con1 that was used to replace the corresponding JFH1 fragment in NS2. For instance, CT1.h refers to the replacement of the *h*elix of JFH1 *T*MS1 by the one of *C*on1 etc. (B) Huh7.5 cells were transfected with constructs specified in the bottom and kinetics of release of infectious particles into culture supernatants were quantified at time points given in the top by TCID_50_ assay. (C) Intracellular and extracellular infectivities were determined 48 h post transfection by using TCID_50_ assay. In panels (B) and (C) representative results of three independent experiments with standard deviations are shown. Background of the assays was determined by using the JFH1-ΔE1E2 mutant in which the region encoding the envelope glycoproteins was deleted and that does not release infectious particles (black line and arrow head). (D) Western blot analysis of HCV proteins expressed in Huh7.5 cells after transfection with constructs specified in the bottom. Cell lysates were prepared 48 h post transfection and proteins were detected by using antibodies with specificities given to the right side of each panel. Beta-actin detected on the same blot was used as internal loading control.

For the reverse genetic studies we used the JFH1 derivative JFH1mut4-6 containing three mutations (V2153A and V2440L in NS5A and V2941M in NS5B) elevating virus titers close to the level of the highly efficient chimera Jc1 (∼10^6^ TCID_50_/ml) without affecting RNA replication [10] (Figure 2A). We chose JFH1mut4-6 for several reasons: first, assembly efficiency of the parental JFH1 genome is very low (∼10^3^ TCID_50_/ml), which precludes its use to select for pseudoreversions; second, the titer enhancing mutations reside in the replicase, thus avoiding possible effects on the structural proteins and the assembly factors p7 and NS2; third, the Jc1 chimera has a cross-over site of two different HCV genomes within NS2 [14], which may confound phenotypes caused by mutations within NS2.

### Intergenotypic swap of TMS domains within NS2 and impact on infectious HCV particle production

In the first set of experiments we generated a panel of NS2 mutants in which individual α-helices of the different TMS of JFH1mut4-6 were replaced by those of the genotype 1b isolate Con1. These ‘helix-swap’ mutations should not affect the secondary structure and thus preserve overall structure, folding and topology of NS2, but disrupt genotype-specific protein-protein interactions. Importantly, since each of the exchanged α-helices of JFH1 and Con1 differ by several aa residues the risk to select for revertants rather than pseudorevertants was very low.

Based on the NMR structures reported earlier [6] and in this study, we constructed 7 helix-swap mutants (aa sequences of affected helices are boxed in Figure 1A): JFH1-CT1.h and CT2.h in which we exchanged the α-helix of TMS1 (aa 11–22 of NS2) or TMS2 (aa 34–46), respectively; JFH1-CT3.h1, JFH1-CT3.h2 and JFH1- CT3.h3 in which the individual short α-helices of TMS3 were exchanged (aa 62–69; 75–85 and 89–97, respectively). In addition, we generated constructs JFH1-CT3.h12 and CT3.h23 in which two short α-helices of TMS3 were exchanged at the same time (aa 62–85 and 75–97, respectively).

As shown in Figure 2B, save for the mutant in which α-helix1 in TMS3 was exchanged (mutant JFH1-CT3.h1), all mutants were profoundly impaired in virus production and infectivity titers were reduced up to 1,000 fold at 72 h after transfection of Huh7.5 cells. This impairment correlated with the degree of sequence conservation between the exchanged helices. Aa sequence alignments revealed that Con1 – JFH1 sequence similarities were in the range of 58–81%, but in case of helix1 of TMS3 similarity was only 37%. Since this helix swap mutant was unaffected in assembly, this region most likely is required for interactions with the membrane and thus genotype independent.

Analysis of intra- and extracellular infectivity revealed that in all cases reduced titers were due to impaired assembly rather than virus release (Figure 2C). With the exception of mutant CT3.h23 producing lower amounts of NS2 assembly defects could not be ascribed to gross alterations of NS2 abundance (Figure 2D). The protein of higher molecular weight detected with mutant JFH1-CT2.h corresponded to uncleaved p7-NS2 arguing for a processing defect of this mutant, which was not the case for all the other mutants. In addition, a distinct product of smaller size (about 16 kDa) was detected on longer exposure (not shown) and this protein most likely corresponds to an N-terminal cleavage product of NS2 designated tNS2 [6] (see below). In summary, these results show that the integrity of the N-terminal MBD of NS2 is important for HCV assembly and that all 3 TMS are required.

### Identification of single amino acid residues in the NS2 membrane binding domain that are essential for HCV assembly

**Figure 3:**
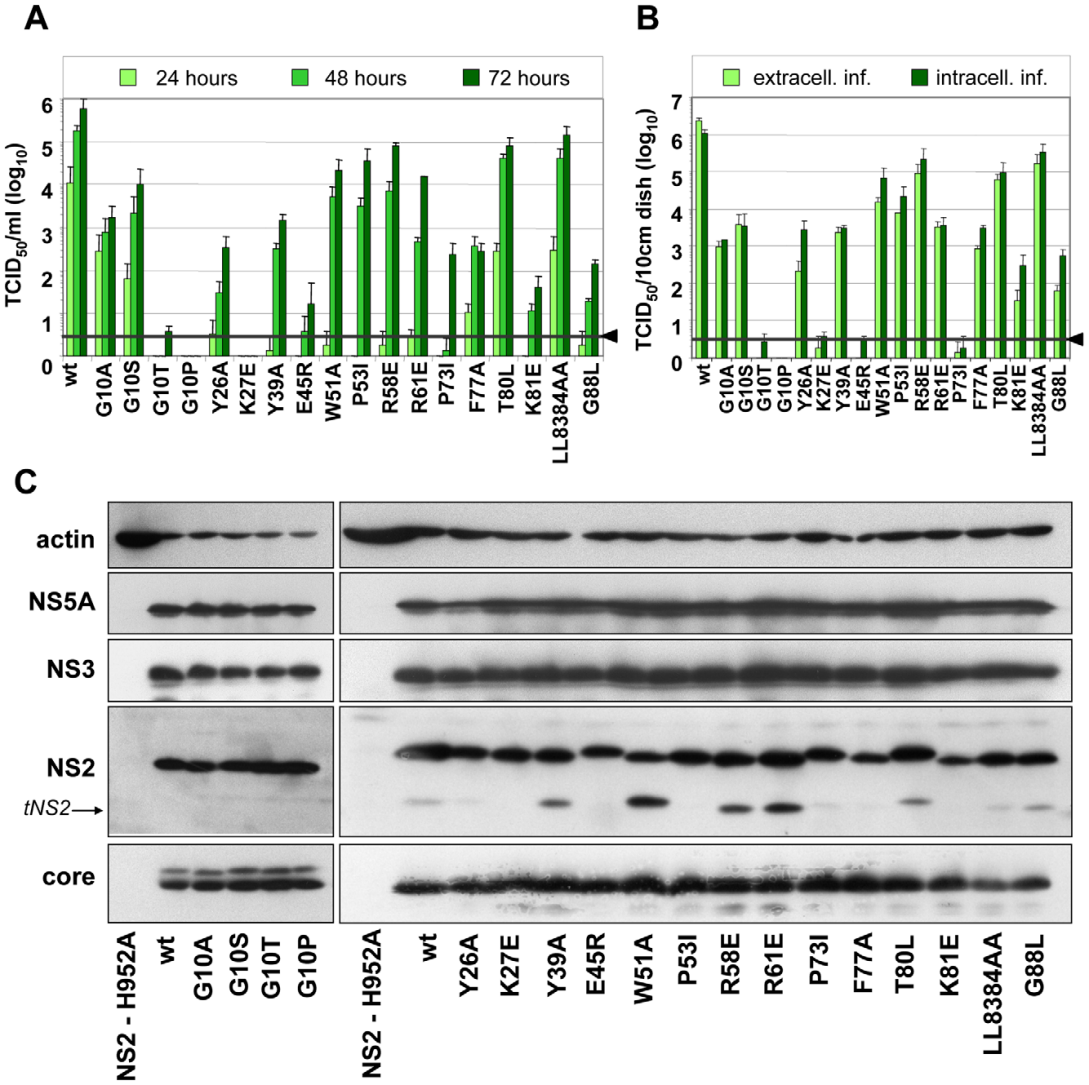
Mutation analysis of the NS2 membrane binding domain identifies amino acid residues that are crucial for efficient virus production. (A) Huh7.5 cells were transfected with JFH1mut4-6 or derivatives thereof containing single point mutations specified in the bottom. Amounts of infectious particles released into the supernatants of cells 24, 48 and 72 h after transfection were determined by TCID_50_ assay. (B) Comparison of intra- and extracellular infectivities obtained with Huh7.5 cells 48 h after transfection. Representative results of three independent experiments with standard deviation are shown in panels (A) and (B). Background of the assays was determined by using JFH1-ΔE1E2 (black line and arrow head). (C) Western blot analysis of viral proteins (core, NS2, NS3 and NS5A) in Huh 7.5 cells 48 h p.t. with constructs specified below the panels. As negative control we used an NS2 mutant unable to replicate because of a mutation blocking the active site of the protease (NS2-H952A; [6]). Beta actin detected on the same blots was used as internal loading control. Wt, wild type; *tNS2*, truncated NS2.

To further narrow down the regions within individual TMS of NS2 that are most crucial for assembly, we generated a panel of single aa substitutions that were designed on the basis of the degree of conservation across the different genotypes, aa size, charge, polarity and hydrophobicity (Figure 1A). Large and hydrophobic aa were replaced by smaller and less hydrophobic ones (Y26P, Y39A, W51A, F77A or LL83-84AA); small aa probably serving as flexible linkers between individual α-helices were replaced by larger aa assumed to reduce flexibility of the N-terminal MBD (G10A/S/T/P, P53I, P73I and G88L); charged aa potentially involved in electrostatic interactions were replaced by aa with opposite charge thus possibly introducing repulsive forces (K27E, E45R, R58E, R61E and K81E); finally, the polar threonine residue at position 80 was replaced by the larger and hydrophobic aa leucine. All mutants were tested for protein expression, kinetic of infectivity release as well as amounts of intra- and extracellular infectivity. The results shown in Figure 3A and B demonstrate that both infectivity release and intracellular infectivity levels were reduced with all mutants, albeit to very different degrees. In agreement with our earlier results demonstrating the important role of the glycine residue at position 10 for virion assembly [6], we found that mutants G10P and G10T did not support virus production and even less drastic alanine or serine substitutions reduced infectivity titers up to 300-fold. Substitutions residing in loop1 that connects α-helix 1 and 2 strongly reduced or completely abolished production of infectious HCV particles (Y26P and K27E, respectively). In contrast, aa substitutions in loop2 (W51A, P53I, R58E and R61E) slowed down the kinetic of infectivity release, which was best detected at 24 h post transfection, whereas intra- and extracellular infectivity titers were reduced only moderately at later time points as compared to the parental construct JFH1mut4-6 (wt).

Alanine substitutions of aromatic aa residues in TMS2 (Y39) or TMS3 (F77) reduced infectivity titers up to 1,000-fold whereas mutations introducing electrostatic repulsion in TMS2 or TMS3 (E45R and K81E, respectively) blocked virus production almost completely. Substitutions targeting the flexible region between helix one and two or helix two and three in TMS3 (P73I and G88L, respectively) strongly reduced infectivity titers (1,000-fold at 72 h p.e.). Surprisingly substitutions affecting the highly conserved polar aa residue at position 80 (T80L) or the two leucine residues at positions 83 and 84 (LL83-84AA) did not give rise to a detectable phenotype. Interestingly, as shown in Figure 1D, residues 80, 83 and 84 are located on one helix side, suggesting that it is not important for protein – protein interactions. In contrast, residues 77 and 81 are located on the opposite side, arguing that this helix side might be involved in interactions.

Western blot analysis of NS2 proteins expressed in cells after transfection with each of the mutants or the parental construct revealed no gross difference in the abundance of this protein and the other HCV proteins arguing for similar replication levels and protein stabilities (Figure 3C). Nevertheless, some variations in abundance of individual HCV proteins were detected such as lower amounts of NS2 in case of K81E and reduced amounts of core protein in case of LL83AA. However, these rather subtle differences are very unlikely to account for the often drastic impairment of HCV assembly. In agreement with an earlier report, ‘truncated NS2’ [6] was detected to variable levels, but its abundance did not correlate with assembly phenotypes.

Given the important role of tryptophan in protein-protein interaction within a membrane [41] and their preferred location at the membrane interface [42], [43], we also analyzed a panel of mutations affecting the two fully conserved W35 and W36 residues. A striking correlation was found between reduction of aromaticity as well as size of residues at these sites and reduction of virus production (data not shown) arguing that the aromatic side chains of W35 and W36 are involved in essential interactions such as membrane tethering of TMS2 via aromatic ring stacking.

### Selection for pseudoreversions rescuing assembly defects caused by mutations within the NS2 membrane binding domain

Having generated a panel of NS2 mutants with a selective assembly defect, and –in some cases- an additional virus release defect (e.g. Y26A; Figure 3B) we wanted to establish a genetic interaction map of NS2. For this purpose we used a cell culture adaptation strategy, which was possible, because the parental construct JFH1mut4-6 that was used for mutagenesis already supports high level virus production and therefore, selection for pseudoreversion would not give rise to undesired mutations enhancing assembly in general [10]. Culture supernatants collected from cells 72 h after transfection with a given NS2 mutant were concentrated and used to infect naive Huh7.5 cells that were passaged 6 times. After 4 additional passages of culture supernatants, they were used to inoculate naïve Huh7.5 cells and virus titers produced therefrom were determined by TCID_50_ assay. In case of mutants with elevated virus titers, cell lysates were used to prepare total RNA, HCV genomes were amplified by RT-PCR and amplicons spanning most of the 5′ NTR up to the middle of NS3 were either directly sequenced or cloned prior to sequence analysis. In the latter case at least two independent clones were analyzed and only mutations conserved between the two cDNA clones were considered in order to discriminate against mutations that might have been introduced by PCR. Pseudoreversions outside the analyzed region, including NS5A, were not considered because we used the JFH1mut4-6 genome that already contained titer-enhancing mutations in NS5A to allow adaptation. Mutations identified by this approach were inserted into the corresponding parental NS2 mutant and replication as well as assembly properties were analyzed by Western blot and TCID_50_ assays. A summary of all pseudoreversions identified in this way along with their degree of titer enhancement is given in Table 1.

**Table 1:** Impact of NS2 mutations and pseudoreversions within and outside of NS2 on production of infectious virus.

construct/mutant	pseudo-reversion	affected protein	Viral titre *	fold titer increase	% of wt titer
**wt**			7.9 10^5^+/−5.6 10^4^	n.a.	100
**CT1.h**			8.0 10^3^+/−1.6 10^3^	n.a.	1.0
	E3D	p7	3.8 10^5^+/−3.9 10^4^	47	48.0
	F14L	NS2	1.5 10^5^+/−7.8 10^4^	19	19.0
	E3D + F14L	p7-NS2	6.9 10^5^+/−1.5 10^5^	85	85.0
	I17M	NS3	2.4 10^3^+/−1.0 10^3^	0.3	0.30
**CT2.h**			4.3 10^2^+/−1.6 10^2^	n.a.	0.05
	N15D	p7	2.4 10^3^+/−2.5 10^2^	5	0.27
	G25R	NS2	1.2 10^5^+/−5.8 10^4^	265	14.4
	N15D + G25R	p7-NS2	1.2 10^5^+/−2.3 10^4^	270	14.7
**CT3.h2**			2.4 10^3^+/−1.0 10^3^	n.a.	0.30
	E151D + I181S	E1	2.9 10^3^+/−7.1 10^2^	1.2	0.36
	K172R	NS2	4.0 10^4^+/−3.8 10^3^	16	4.9
**CT3.h3**			1.4 10^3^+/−7.9 10^2^	n.a.	0.18
	T21A	NS2	2.5 10^4^+/−1.0 10^3^	17	3.0
*Single AA substitution in TMS1*					
G10A			1.3 10^3^+/−1.3 10^2^	n.a.	0.16
	A10G	NS2	n.a.	n.a.	n.a.
G10S			5.8 10^3^+/−2.9 10^3^	n.a.	0.73
	T23N	NS2	1.3 10^6^+/−7.5 10^5^	227	166
G10T			2.4 10^0^+/−1.0 10^0^	n.a.	<0.01
	T23N **	NS2	1.9 10^2^+/−9.5 10^1^	100	0.03
G10P			n.d.	n.a.	n.a.
	T23N **	NS2	1.4 10^2^+/−8.3 10^1^	n.a.	0.02
*Single AA substitution in TMS2*					
W35A			*n.d.*	n.a.	n.a.
	Q221L **	NS3	3.4 10^2^+/−1.0 10^2^	n.a.	0.04
W35L			n.d.	n.a.	n.a.
	Q221L **	NS3	1.9 10^3^+/−7.6 10^2^	n.a.	0.24
W35F			8.7 10^1^+/−1.4 10^1^	n.a.	0.01
	Q221L	NS3	1.2 10^5^+/−6.6 10^4^	1,400	14
W36A			n.d.	n.a.	n.a.
	Q221L **	NS3	5.8 10^4^+/−1.3 10^4^	n.a.	7.3
W36L			3.2 10^0^+/−6.0 10^-1^	n.a.	<0.01
	Q221L	NS3	1.3 10^5^+/−6.7 10^4^	38,200	16.5
W36F			2.7 10^2^+/−7.4 10^1^	n.a.	0.03
	Q32R	NS2	1.2 10^5^+/−6.6 10^4^	450	15.3
Y39A			1.0 10^4^+/−7.5 10^3^	n.a.	1.3
	G25R	NS2	1.2 10^5^+/−1.2 10^4^	11	13.9
E45R			3.2 10^0^+/−4.8 10^-1^	n.a.	<0.01
	R45G	NS2	2.9 10^5^+/−3.0 10^4^	87,900	36.7
*Single AA substitution in TMS3.2*					
P73I			1.4 10^2^+/−6.3 10^1^	n.a.	0.02
	I73S	NS2	4.3 10^5^+/−1.9 10^5^	3,100	55.1
F77A			4.1 10^2^+/−2.4 10^2^	n.a.	0,05
	Y215S	E2	n.d.	n.a.	n.a.
	V341A	E2	4.3 10^4^+/−4.0 10^2^	100	5.4
K81E			3.9 10^1^+/−2.3 10^1^	n.a.	<0.01
	E81K	NS2	n.a.	n.a.	n.a.
K81D			3.5 10^1^+/−1.5 10^1^	n.a.	<0,01
G88L			1.4 10^2^+/−4.1 10^1^	n.a.	0,02

*72 h post transfection.

**artificial insertion of adaptive mutation; not detected during selection for pseudoreversion.

n.d.: no detectable infectivity (detection limit 2x10^0^ TCID_50_/ml).

n.a.: not applicable.

**Figure 4:**
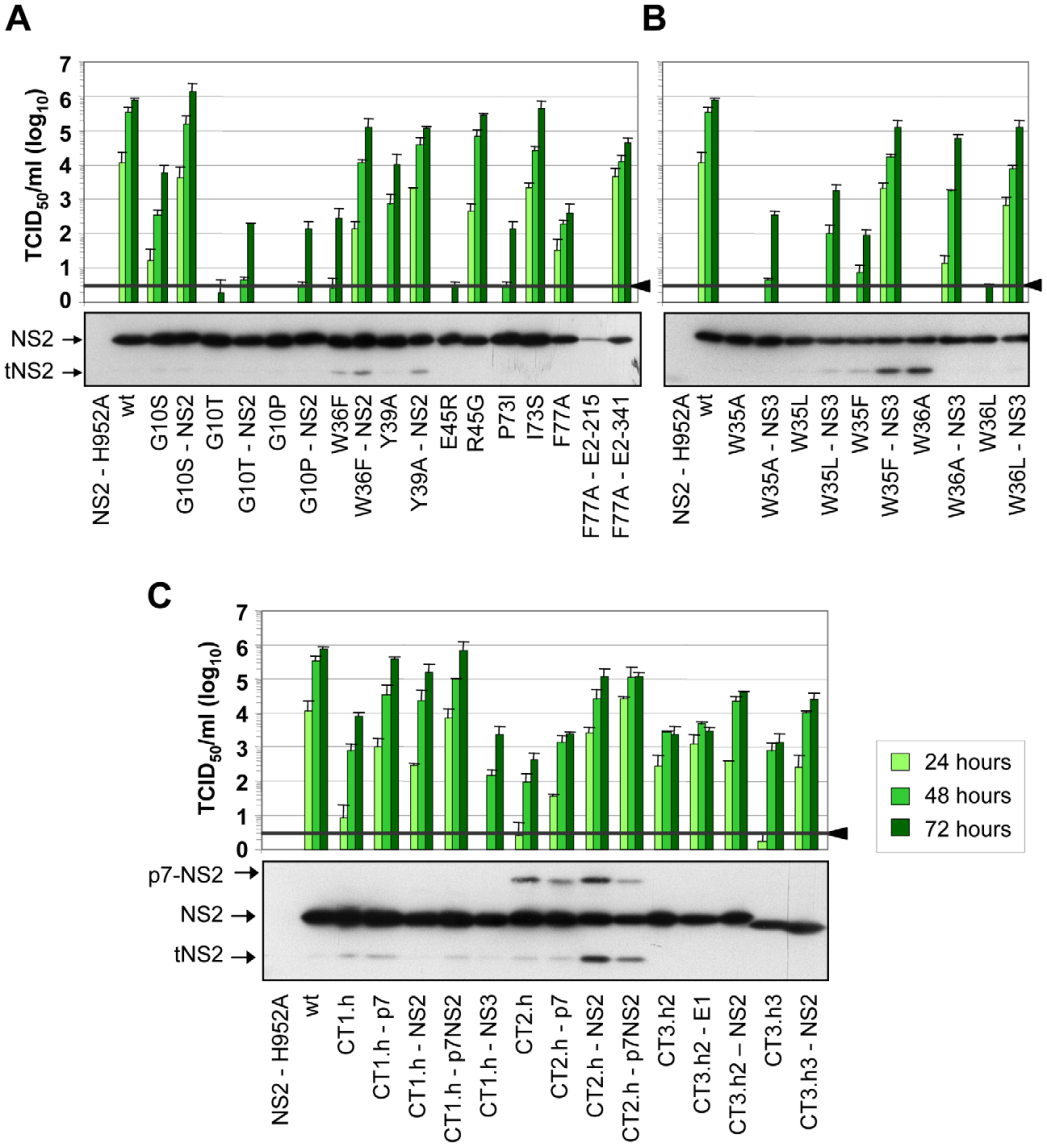
Functional analysis of pseudoreversions within and outside of NS2 for their capability to restore virus particle production. JFH1mut4-6 mutants carrying either single aa subtitutions (panel A and B) or helix swaps (C) causing a profound reduction of virion production were used for insertion of pseudoreversions that are specified in Table 1. Parental NS2 mutants and corresponding pseudorevertants were transfected into Huh7.5 cells and kinetic of virus production was determined by TCID_50_ assay at time points specified in the top. A representative result of two independent experiments with standard deviations is shown in each panel. Background of the assays was determined by using JFH1-ΔE1E2 (black line and arrow head). Shown below each panel are the NS2 expression levels of mutants and the corresponding pseudorevertants as determined by Western blot using lysates of Huh7.5 cells prepared 48 h after transfection. A more comprehensive analysis of additional viral proteins is shown in Supplementary Figure S4.

Several assembly deficient mutants (G10T, G10P, K81D, G88L and JFH1-CT3.h23) could not be adapted, because the virus was rapidly lost during cell passages suggesting that the genetic barrier was too high and assembly impairment too strong. Nevertheless, for most mutants we could select for pseudoreversions with the exception of G10A and K81E where reversion to wild type occured, which was achieved by just one nucleotide substitution. Since we had inserted in addition a silent nucleotide exchange in the subsequent codon that was retained in the selected virus, we could rule out a contamination with wild type virus. All of the other adapted mutants contained pseudoreversions. They resided primarily within NS2 and two ‘classes’ of pseudoreversions could be discriminated: first, those residing at the same position as the primary mutation, but with a different substituting aa residue; second, pseudoreversions at a different site than the primary mutation. Pseudoreversions belonging to the first class (R45G and I73S) enhanced virus production 88,000- and 3,100-fold and thus back to the level of the parental genome JFH1mut4-6 (Figure 4A and Table 1). Pseudoreversions belonging to the second class (double mutants G10S-T23N, W36F-Q32R and Y39A-G25R) increased infectivity titers ∼220-, 450- and 10-fold, respectively, with G10S-T23N also reaching wild type levels (Figure 4A and Table 1). For this reason the T23N substitution was also combined with mutants G10T and G10P, but titer increase was very moderate (Figure 4A).

In addition to pseudoreversions in NS2, one particular mutation was identified in NS3 (Q221L) that rescued infectivity titers of the W35F and the W36L mutants by 1,400- and 38,000-fold (Figure 4B). This NS3 mutation has previously been described as a general titer enhancing substitution [25], [27]. For this reason we inserted the Q221L mutation into mutants W35A, W35L and W36A that are completely defective in assembly and that could not be adapted with our approach. As shown in Figure 4B, infectivity titers were enhanced in all cases corroborating the more general assembly enhancing phenotype exerted by this particular NS3 mutation. Moreover, the same mutation was also capable to rescue virus production of several NS2 mutants even in trans (supplementary Figure S2). Although in this case rescue efficiency was lower as compared to direct insertion of the mutation into the NS2 mutant genome, this observation suggests that a genetic separation of the replication and assembly function of NS3 is possible.

In case of the F77A mutation residing in TMS3.2 of NS2 two mutations located in E2 could be selected: Y215S located in the ectodomain and V341A in the N-terminus of the TMS of E2 (Table 1). When we tested these substitions in the context of the parental NS2 mutant we found that Y215S completely abrogated infectious virus production concomitant with a strong reduction in intracellular NS2 amounts (Figure 4A). However, the V341A substitution enhanced virus titer ∼100-fold arguing for a (direct or indirect) interaction between these two proteins for efficient particle production.

A more complex pattern of mutations was found upon selection for pseudoreversions in case of helix-swap mutants JFH1-CT1.h, JFH1-CT2.h, JFH1-CT3.h2 and JFH1-CT3.h3 (Table 1 and Figure 4C). For CT1.h, we found E3D residing in the N-terminal helix of p7 [16], F14L in TMS1 of NS2 and I17M in the membrane-binding amphipathic helix α0 of the NS3 protease domain. When inserted into the parental virus, strongest rescue of assembly was found with the p7 mutation and infectivity titers were further increased 10-fold at 24 h or 2-fold at 72 h p.t. when combined with the NS2 pseudoreversion (CT1.h - p7NS2). In contrast, the NS3 mutation had a slightly negative effect (CT1.h-NS3-I17M). For the JFH1-CT2.h mutant we detected two pseudoreversions residing in the turn connecting the N-terminal helix and the TM1 helix of p7 (N15D) [16] and the loop connecting TMS1 and TMS2 of NS2 (G25R). In this case, the mutation in NS2 rescued HCV assembly strongest (∼265-fold) whereas the p7 mutation had a very moderate effect and in combination with the NS2 mutation did not enhance virus titers further. In case of pseudoreversions detected with helix-swap mutants JFH1-CT3.h2 and JFH1-CT3.h3, the only titer enhancing mutations were found in NS2 (K172R, T21A) with T21A arguing for an interaction between TMS1 and TMS3 of NS2. The double mutation identified in E1 of JFH1-CT3.h2 construct (E151D/I181S) had no effect.

For all tested single aa mutants and helix-swap mutants and their corresponding pseudorevertants, the enhancement of infectivity titers in cell culture supernatants correlated with increased amounts of intracellular infectivity showing that the pseudoreversions rescued primarily assembly rather than virus release (supplementary Figure S3). Moreover, with the exception of the F77A-E2-Y215S double mutant, amounts of NS2 as well as NS5A, NS3 and core protein were not grossly altered (Figure 4 and supplementary Figure S4, respectively) suggesting that overall protein stabilities were not profoundly affected by the mutations. We note however that for mutant F77A already producing somewhat lower amounts of NS2 as compared to the wildtype, NS2 abundance was reduced much more by the Y215S substitution in E2. Moreover, in case of helix-swap mutant CT2.h and the corresponding pseudorevertants cleavage between p7 and NS2 was impaired (Figure 4C). Interestingly, even in case of rescue mutant CT2.h-NS2, the substitution in NS2 enhancing virus production about 250-fold does not affect the amounts of this uncleaved precursor arguing that assembly competence of this mutant is restored in a manner that still allows delayed p7-NS2 cleavage, thus compensating e.g. an impaired p7-NS2 interaction (see below).

### Construction and characterization of fully functional JFH1-derived genomes encoding an N-terminally tagged NS2 protein

**Figure 5:**
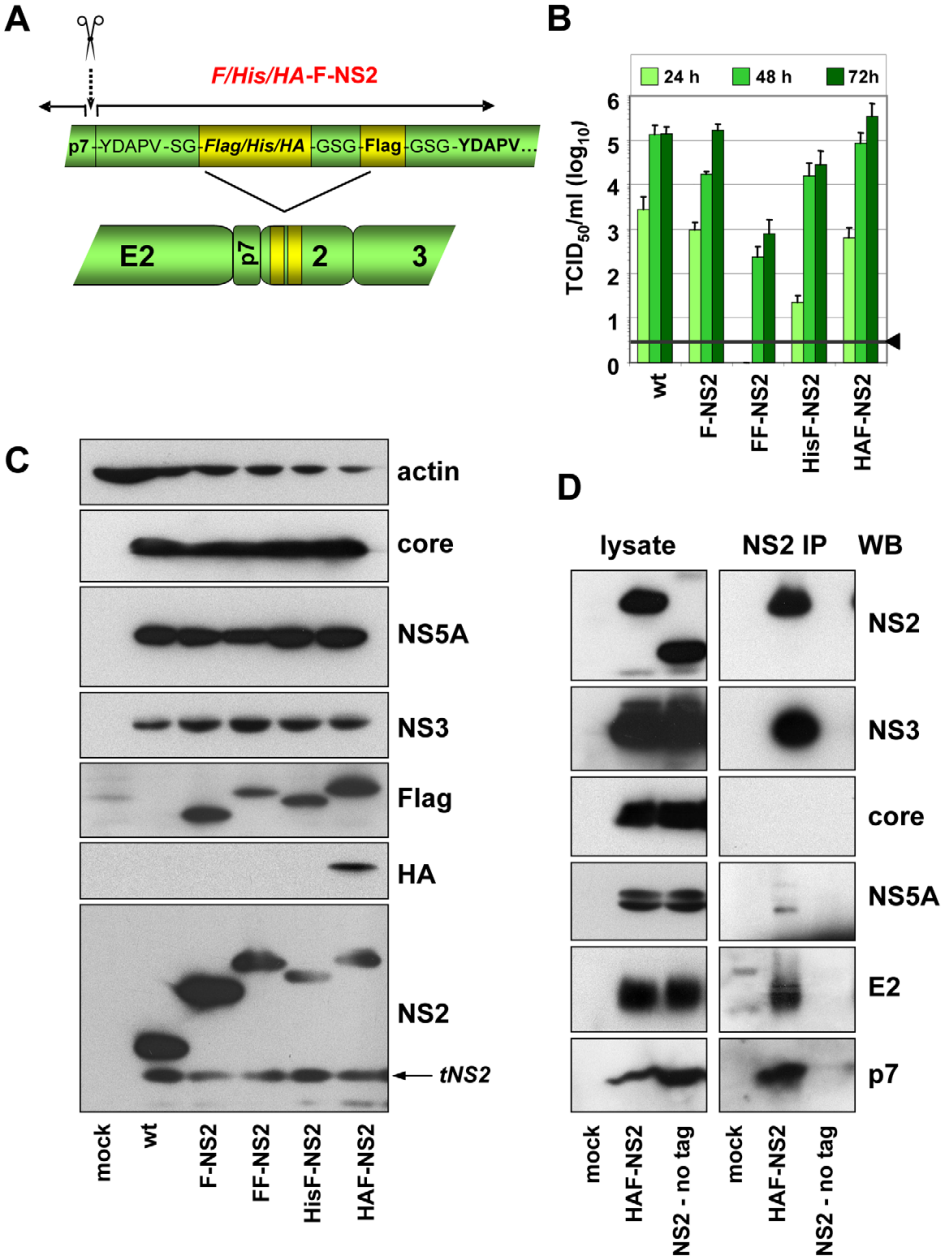
Construction and functional characterization of JFH1 genomes encoding an N-terminally tagged NS2 protein. (A) Schematic representation of the JFH1mut4-6 constructs in which the N-terminus of NS2 was fused to either the Flag-tag (F) or two copies of this tag (FF) or a hexahistidin-Flag fusion tag (HisF) or a HA-Flag fusion tag (HAF). The cleavage site between p7 and NS2 is indicated with a dotted arrow and the scissor; aa sequences of the duplicated N-terminal 5 aa residues and the Gly-Ser linkers are displayed. (B) Kinetic of virus production attained with JFH1mut4-6 derivatives depicted in panel (A) was determined by transient transfection of Huh7.5 cells in parallel with the parental genome (wt). A representative result of three independent experiments with standard deviations is shown. Background of the assays was determined by using JFH1-ΔE1E2 (black line and arrow head). (C) Expression of HCV proteins was determined by Western blot analysis 48 h after transfection using antibodies specified in the right. (D) Interaction of NS2 with structural and nonstructural proteins. JFH1mut4-6 without a tagged NS2 (wt) or a derivative thereof with the HA- and Flag-tag fused to the N-terminus of NS2 (HAF-NS2) were transfected into Huh7.5 cells. Seventytwo hours later cells were harvested and lysates were used for immunoprecipitation with an HA-specific antibody. After extensive washing, immunocomplexes were analyzed by electrophoresis into a Tris-Tricine gel and subsequent Western blot using antibodies given in the right of each panel (NS2 IP). Ten percent of the input used for immuno-capture was analyzed in parallel (left panels). Mock-transfected cells served as negative control of Western blot and the wt genome lacking the HA-tag was used as specificity control for immunoprecipitation.

Although in most cases, selection for pseudoreversion resulted in compensatory mutations within NS2 itself, we also identified pseudoreversions in E2, p7 and NS3. In case of helix-swap mutant CT1.h, the E3D substitution in p7 restored almost wild type infectivity titers (Table 1). Likewise, the assembly defect of mutant W35F in NS2 was compensated by Q221L in NS3 and NS2 mutant F77A was compensated by the V341A substitution in E2. These results suggested that NS2 might interact (directly or indirectly) with each of these proteins. To support this assumption by pull-down assays, we first generated a fully functional JFH1-derivative with a tagged NS2 protein suitable for efficient immunoprecipitation and allowing capture of NS2 independent from any mutation that might affect recognition with the NS2-specific antibody. To this end we constructed a series of mutants in which NS2 was fused N- or C-terminally with several tags such as the FLAG-, hexa-histidine (His)- or hemagglutinin (HA)-tag. All genomes with C-terminal fusions no longer supported HCV particle production (data not shown). Moreover, when we tried to select for titer enhancing pseudorevertants of assembly deficient NS2-tagged mutants, in all cases the tag was partially or completely deleted (not shown). In contrast, viable mutants were obtained with N-terminally tagged NS2 versions in which the first 5 codons of NS2 were duplicated upstream of the heterologous sequence that was composed of a single copy of the tag and a linker sequence encoding for Gly-Ser-Gly preceeding NS2. In addition, variants were generated with a second insertion of the tag sequence to increase efficiency of immunoprecipitation (Figure 5A). Analysis of the kinetics of virus production revealed that both the single Flag-tagged (F-NS2) and the HA-Flag-double tagged variant (HAF-NS2) produced amounts of intra- and extracellular virions that were comparable to the parental genome JFH1mut4-6 (data not shown and Figure 5B). In contrast, the variants with the tandem Flag-tag (FF-NS2) or the His-Flag-tag combination (HisF-NS2) produced lower amounts of extra- and intracellular infectious particles arguing for an assembly defect. Western blot analysis revealed comparable replication of all constructs and no defect of polyprotein processing was detected (Figure 5C). The sizes of the various NS2 proteins and their immunoreactivities confirmed that the tag(s) remained fused to mature (fully processed) NS2.

### Interaction of NS2 with E2, p7, NS3 and NS5A as determined by co-immunoprecipitation

Taking advantage of these assembly-competent tagged NS2 constructs, we selected JFH1mut4-6HAF-NS2 to determine NS2 interactants. Huh7.5 cells were transfected with this construct and NS2-containing immunocomplexes captured from lysates that were prepared 72 h p.t. were analyzed by Western-blot for coprecipitation of core, E2, p7, NS3 and NS5A. Specificity of immunoprecipitation and Western blot analysis was determined by using the parental JFHmut4-6 construct that lacked the N-terminal NS2-tag. The results in Figure 5D show that the tagged NS2 protein co-precipitated with E2, p7, NS3 and NS5A, but not with core. In contrast, no signal was found in case of the non-tagged genome inspite of comparable amounts of viral proteins in cell lysates, demonstrating specificity of these co-precipitations.

**Figure 6:**
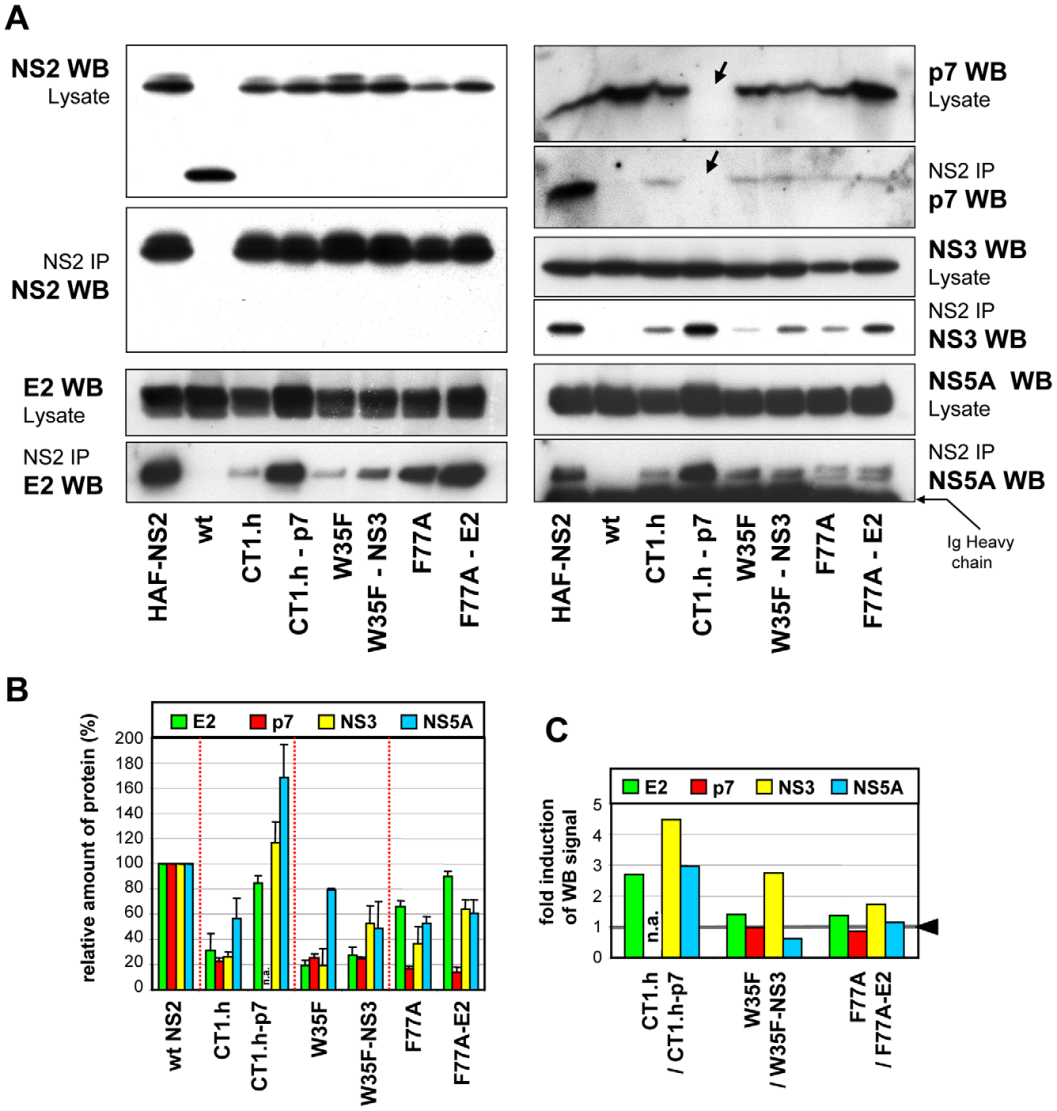
NS2 mutations and pseudoreversions and their impact on NS2 interaction with other viral proteins. JFHmut4-6 (wild type without tag; wt), JFHmut4-6HAF-NS2 with the HA-Flag double tag or helix swap mutants and double mutants derived from this construct were transfected into Huh7.5 cells and harvested 72 h later. (A) Cell lysates were used for immunoprecipitation (IP) with an HA-specific antibody and immunocomplexes were analyzed by Western blot (WB) as specified to the left and right of each panel. For comparison, 10% of the lysate used for the IP was analyzed in parallel. The wt genome lacking the HA-tag was used as specificity control for immunoprecipitation. Note that in case of the p7 mutant the pseudoreversion (E3D) resides in the epitope recognized by the p7-specific antibody and thus this p7 protein is not detected (labeled with an arrow). (B) Western blot signals of proteins in immunocomplexes from two independent experiments were quantified by densitometry scanning and normalized to the amounts of the respective viral protein present in the cell lysate. Values obtained with the wild type genome were set to 100%. (C) Relative enhancement of co-immunoprecipitation of NS2 with HCV proteins specified in the top by pseudoreversions. Values obtained with the corresponding NS2 mutant in the absence of the pseudoreversion were set to 1 (highlighted with the grey line and the black arrow head) and used for normalization of the values measured with the pseudorevertants.

To analyze whether the pseudoreversions in E2, p7 and NS3 affect the NS2 interaction pattern, we chose those NS2 mutants for which infectivity titers were enhanced by a pseudoreversion outside of NS2: CT1.h and CT1.h-p7-E3D; W35F and W35F-NS3-Q221L; F77A and F77A-E2-V341A. Lysates of all samples harvested 72 h after electroporation together with positive and negative controls were subjected to HA-specific pull-down and immunocomplexes were analyzed by Western blot (Figure 6A). Pull-down efficiencies were quantified by densitometry scanning and normalized to protein amounts detected in the corresponding cell lysate (Figure 6B); based on this quantification fold enhancement of coimmunoprecipitation achieved by the pseudoreversion was determined (Figure 6C).

For all mutants, NS2 interaction with the other viral proteins was reduced, but to very different extents. Most pronounced was the impairment of NS2 interaction with E2, p7 and NS3, whereas interaction with NS5A was less affected. Importantly, the E3D pseudoreversion in p7 introduced into CT1.h enhanced interaction with E2 and NS3 back to wild type levels correlating well with the rescue of assembly competence of this helix-swap mutant. Unfortunately this pseudoreversion disrupted the epitope recognized by the p7-specific antibody and therefore, the degree of coprecipitation of this p7 with NS2 could not be determined. Interaction of NS2 with NS5A was elevated even above the wild type level (Figure 6B, C). As expected, the Q221L pseudoreversion in NS3 introduced into the W35F NS2 mutant increased NS2 – NS3 interaction, but surprisingly had little or no effect on NS2 interaction with E2 or p7, respectively, and even a negative effect on interaction with NS5A (Figure 6B, C). The pseudoreversion in the TMS of E2 (V341A) introduced into the F77A mutant moderately enhanced interaction of NS2 with E2 and NS3, but no significant enhancement of interaction with p7 and NS5A was detected. Interestingly, NS2 containing this F77A substitution coprecipitated with both phospho-variants of NS5A to the same extent, whereas all other NS2 proteins tested preferentially interacted with the basal phosphorylated form p56 (Figure 6A). This phenotype of the F77A mutant was not altered by the adaptive mutation residing in E2.

### Subcellular localization studies of NS2

**Figure 7:**
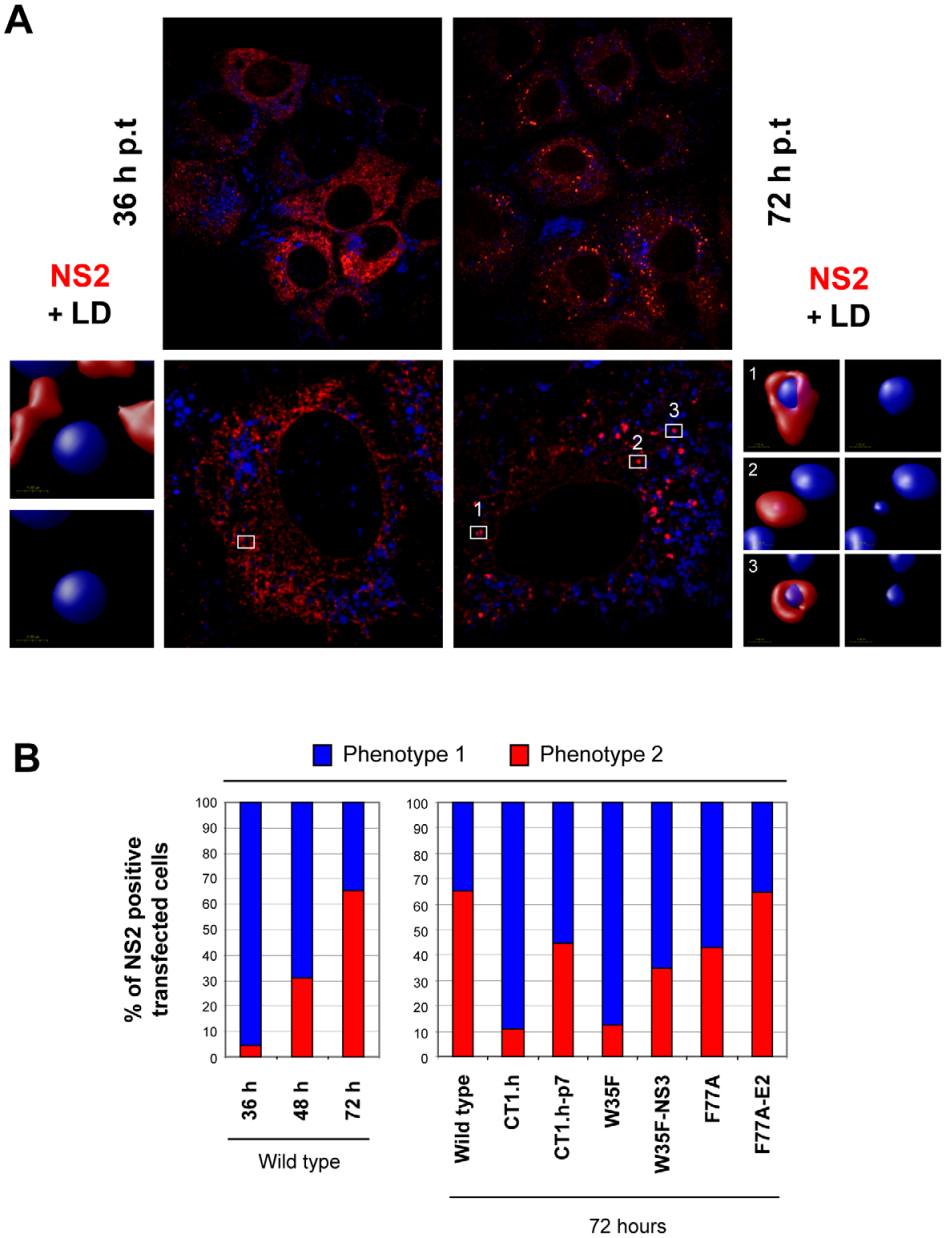
Time-dependent change of NS2 subcellular localization. Huh7-Lunet cells were transfected with JFH1mut4-6 HAF-NS2 and 36, 48 and 72 h later, NS2 and LDs were detected by using an HA-specific antibody (red) or BODIPY staining (blue), respectively. (A) Representative images of cells 36 and 72 h p.t. (left and right half, respectively). An overview is shown in the top, a magnification of a single cell from a different view field below. Boxed areas are enlarged in the smaller panels to the left and right, respectively. Enlargements show 3D reconstructed images created with the Volocity 5.3 software. In case of the 72 h value 3 representative structures are displayed. The staining pattern shown for the 36 h time point and in panel 2 for the 72 h time point corresponds to phenotype 1. (B) Huh7-Lunet cells were transfected with the wild type (left panel) or given NS2 mutants (right panel) and analyzed as described above. Shown are the ratios of phenotype 1 and 2. In case of the wild type values determined at different time points after transfection are shown, in case of the mutants only the values measured 72 h p.t. For each construct, 180 cells were analyzed.

To support and extend the interaction patterns described above with an alternative assay we performed colocalization studies of NS2 with structural and other nonstructural proteins. In the initial set of experiments, we determined the subcellular localization of NS2 (Figure 7A) and observed a profound change from a reticular ER staining pattern 36 h post transfection (NS2 colocalization with the ER marker PDI is not shown) to a strong punctate NS2 stain accumulating in close proximity of LDs 72 h post transfection. By counting ∼200 cells we defined two phenotypes, based on the number of LDs with NS2 accumulation: phenotype 1 with less than 10 NS2-positive LD structures per cell and phenotype 2 with more than 10. A time-dependent increase of phenotype 2 was also observed although the overall percentage was lower, which was probably due to lower replication as compared to RNA transfection (supplementary Figure S5).

The functional relevance of these two phenotypes is supported by the analysis of the NS2 mutants and their respective pseudorevertants (Figure 7B). We found that NS2 decorated LDs were much less frequent in an assembly deficient mutant and even 72 h after transfection the majority of NS2 was localized at the ER. Importantly, upon insertion of the corresponding pseudoreversion a shift back to phenotype 2 representing higher abundance of NS2 ‘positive’ LDs 72 h p.t. was detected (Figure 7B).

**Figure 8:**
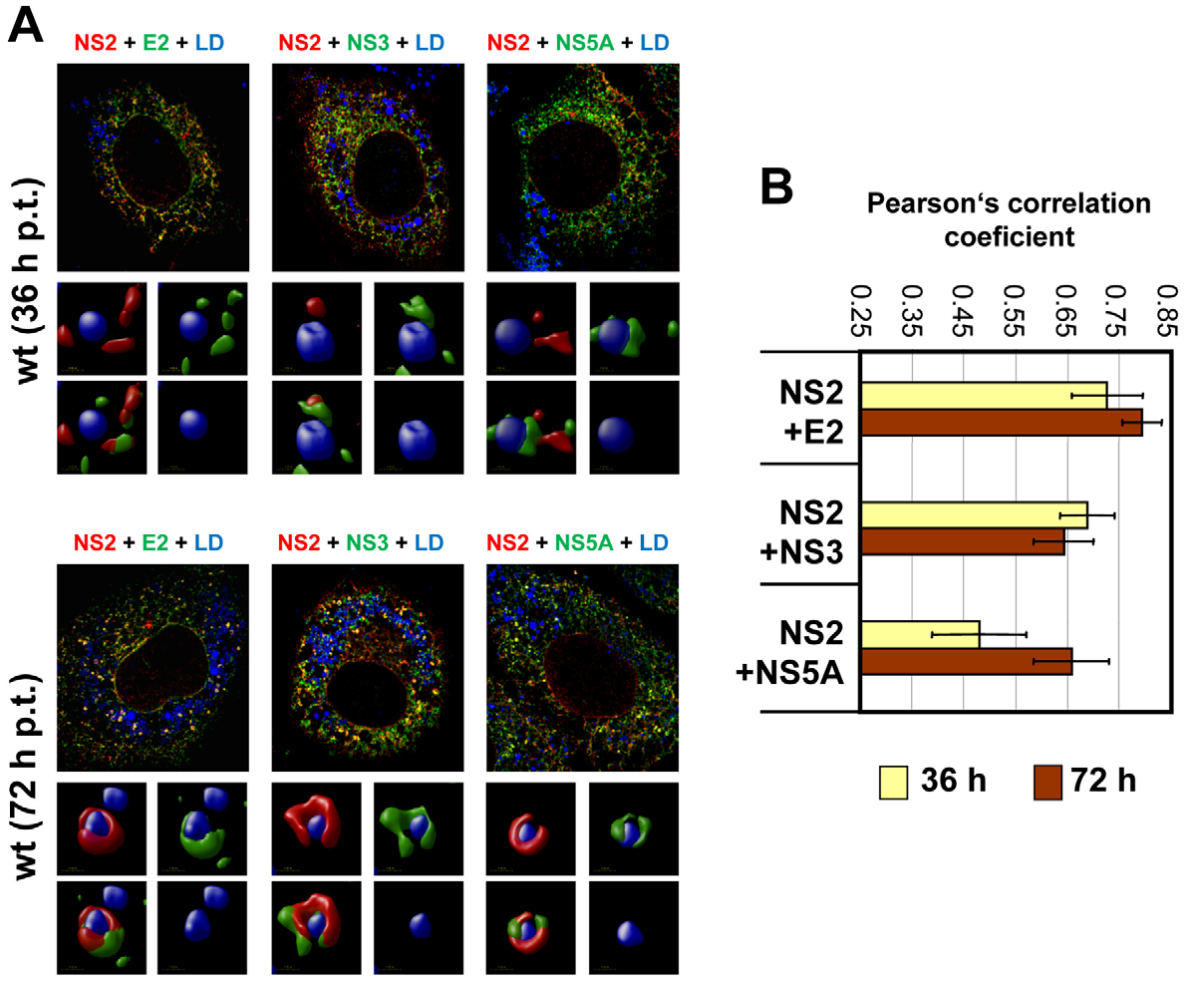
Colocalization of NS2 with structural and nonstructural proteins and recruitment to LDs. (A) Huh7-Lunet cells were transfected with JFHmut4-6-HAFNS2 (wild type, wt) and 36 h or 72 h later NS2 was detected with an HA-specific antibody (red). E2, NS3 or NS5A were detected with mono-specific antisera and LDs were labeled with BODIPY (blue). Pictures were deconvolved by using the Huygens Essential 3.5 software and a theoretical point spread function. The small inserts in the bottom of each panel represent 3D reconstructed enlargements of each corresponding image created with the Volocity 5.3 software. (B) The degree of colocalization was quantified by determining Pearson's correlation coefficients. For each sample 50 cells were quantified by using the ImageJ software. The background of the assay (0.25) was determined by analyzing cells co-expressing cytosolic RFP and HA tagged NS2; the maximum of colocalization (0.85) was established by using double staining of E2 with two different antibodies.

To determine whether other viral proteins might be recruited to LDs in an NS2-dependent manner we performed colocalization studies. As shown in Figure 8A, at each analyzed time point we found a striking colocalization of NS2 and E2 in case of the wild type, consistent with the coimmunoprecipitation results. In addition, we detected a strong accumulation of both proteins around LDs 72 h p.t. (Figure 8A). A similar pattern, but less colocalization as determined by Pearson's correlation coefficient, was found for NS2 with NS3 (Figure 8B). Interestingly, a lower degree of colocalization of NS2 and NS5A predominated 36 h p.t. and NS5A localized in close proximity of LDs independent of NS2. Accumulation of NS2 around LDs at the later time point coincided with increased NS2-NS5A colocalization at these sites. No significant colocalization was detected between NS2 and core protein at LDs. However, a small amount of core colocalized with NS2 in a reticular, presumably ER-derived compartment (supplementary Figure S6). Attempts to detect p7 by immunofluorescence were not successful with the available antibodies and insertion of tags into p7 very much impaired assembly (not shown). Therefore, p7 – NS2 colocalization could not be studied.

**Figure 9:**
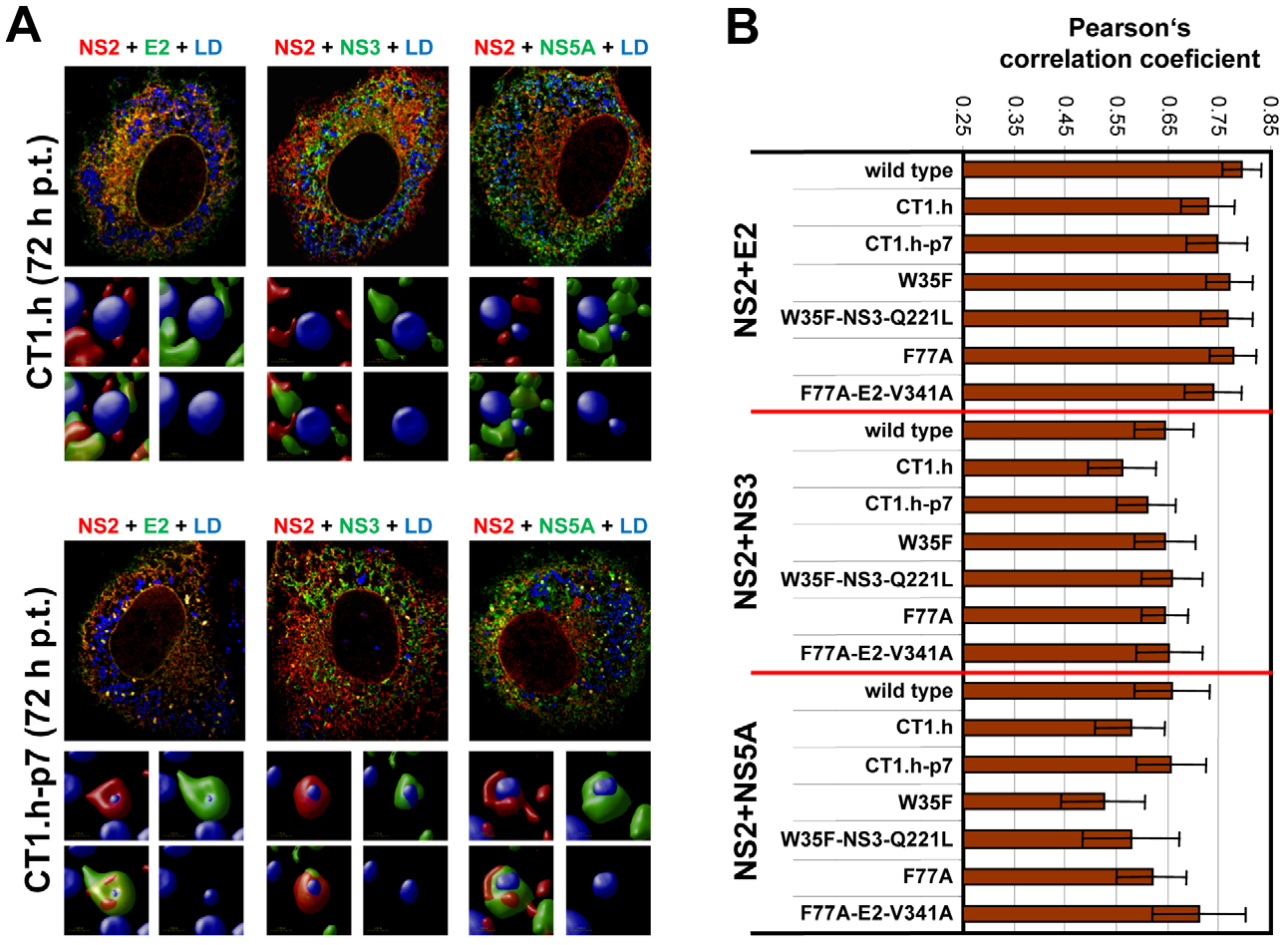
Impact of NS2 mutations and the corresponding pseudoreversions on colocalization with structural and nonstructural proteins and accumulation around LDs. (A) Huh7-Lunet cells were transfected with helix-swap mutation CT1.h or its corresponding pseudorevertant (CT1.h-p7) and NS2 colocalization with E2, NS3 and NS5A as well as their accumulation around LDs was determined by immunofluorescence. Cells were analyzed 72 h post transfection as described in Figure 8. The small inserts in the bottom of each panel represent 3D reconstructed enlargements of each corresponding image created with the Volocity 5.3 software. (B) Colocalization of NS2 with E2 or NS3 or NS5A was quantified by determining Pearson's correlation coefficients providing a measure for the relative degree of co-localization of proteins. For each sample 50 cells were quantified by using the ImageJ software. For further details see legend to Figure 8.

Given the most pronounced loss of NS2 accumulation around LDs (i.e. low frequency of phenotype 1) with mutant CT1.h we determined for this construct and the corresponding pseudorevertant NS2 colocalization with E2, NS3 and NS5A as well as HCV protein accumulation at LDs. For the parental NS2 mutant we found that E2 no longer localized to LDs and localization of NS3 to these sites was strongly impaired (Figure 9A). However, recruitment of these viral proteins to LDs and strong colocalization at LDs was restored by the pseudoreversion in p7 (E3D; Figure 9A, lower panel). In contrast, NS5A was recruited to LDs independent from NS2 and NS2 – NS5A colocalization was also restored by this pseudoreversion.

When analyzing a larger panel of NS2 mutants and their corresponding pseudorevertants for colocalization of these HCV proteins in a quantitative manner (Figure 9B) we found for CT1.h a slight reduction of NS2 colocalization with E2, NS3 and NS5A that was partially restored by the pseudoreversion residing in p7 (CT1.h-p7). In case of NS2 mutants W35F and F77A only NS2 colocalization with NS5A was impaired, but restored by the corresponding pseudoreversion in NS3 or E2 (W35F-NS3-Q221L or F77A-E2-V341A, respectively). In contrast, colocalization of NS2 with E2 or NS3 was unaffected by these NS2 mutations (Figure 9B). This result could be explained by the fact that the mutations in NS2 might impair accumulation around LDs and thus would lead to accumulation of NS2 at ER membranes where also the majority of E2 and NS3 reside. Therefore, colocalization of these NS2 proteins with E2 and NS3 (at the ER membrane) might be strong. In contrast, NS5A is recruited to LDs independent of NS2 and therefore, NS2 mutations that no longer are recruited to LDs might have lower colocalization rate that would be restored by the pseudoreversion that rescues ‘LD targeting’ of NS2.

## Discussion

The important role of NS2 for HCV assembly has been shown by several earlier reports [5], [6], [25]–[27]. However, the way this protein contributes to virion formation remains enigmatic. We addressed this question by using several complementary approaches including the structural analysis of the MBD, reverse and forward genetic analyses and a combination of coimmunoprecipitation and subcellular localization studies. Our results provide evidence that NS2 recruits the envelope glycoproteins (presumably in conjunction with p7) and probably also NS3 to LDs and serves as a key organizer of HCV assembly by participating in multiple protein - protein interactions required for virion formation. The implications of these results are discussed in the following.

### Topology model of NS2 MBD

By using NMR of synthetic peptides we solved the secondary structures of TMS2 and 3 and propose a membrane topology model of the overall N-terminal MBD (Figure 1D). This model supports and very much extends an earlier report [28] and proposes 3 transmembrane α-helices, connected by flexible loop regions. While TMS1 and 2 consist of one α-helix, TMS3 is composed of three. Each of TMS1 - 3 is capable to mediate membrane association on its own. To determine whether individual helices within TMS3 are sufficient for membrane targeting we analyzed subcellular localization patterns of NS2-GFP fusion proteins comprising NS2 aa residues 60–88 or 74–99. However, these proteins displayed a predominantly diffuse fluorescence signal, arguing that all 3 helices of TMS3 are required for membrane targeting (J.G. and D.M., unpublished).

**Figure 10:**
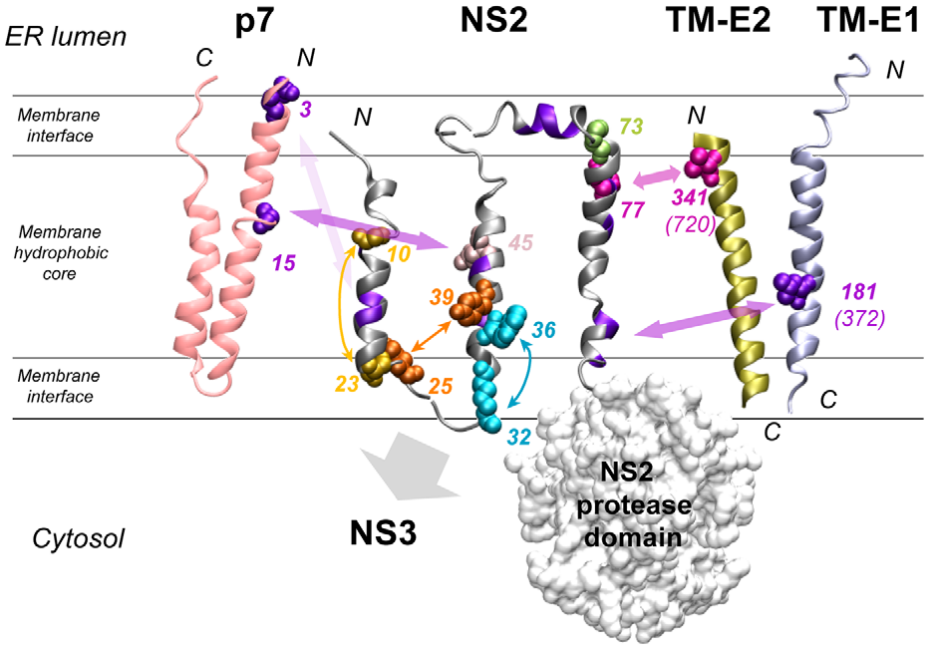
Summary of interactions within NS2 and with HCV proteins as deduced from the genetic analysis. The structural models of NS2 (this study), p7 [16], the transmembrane domain of E1 (TM-E1;[68]) and E2 (TM-E2; theoretical model) are represented as ribbon. The phospholipid bilayer is indicated tentatively and schematically. Only the side chains of aa encoded by pseudoreversions are shown with spheres correspondíng to van der Waals radia. Residues likely involved in intra- or inter-molecular interactions are indicated with the same color and connected with double arrows. Interactions within and outside of NS2 are represented by small and large double arrows, respectively. The backbone of aa residues with low similarity identified in helix-swap mutants and potentially involved in inter-helix interactions are colored violet as in Figure 1D. The large gray arrow indicates the region of NS2 likely involved in interaction with NS3. Residues colored in pink-gray (position 45) and light green (position 73) correspond to pseudoreversions at the very same site as the original NS2 mutation. Numbering of altered residues in TM-E1 and TM-E2 correspond to JFH1 and numbers in parenthesis correspond to the H77 reference polyprotein. The molecular models of TM-E1 and TM-E2 were constructed by using the Swiss Model server facilities (http://www.expasy.ch/swissmod/). The figure was generated on the basis of the structure coordinates by using the Visual Molecular Dynamics program and rendered with POV-Ray.

The model of three TMS is consistent with homo-intramolecular TMS interactions revealed by the pseudoreversions indicating that TMS1 interacts with TMS2 and TMS3. The model is also consistent with hetero-intermolecular interactions by which TMS1 and TMS2 could interact with p7 whereas TMS3 could interact with the TMS of E1 and E2 (Figure 10). Moreover, the fact that point mutations in the long and variable connecting loop between TMS2 and TMS3 had no effect on virus production is in keeping with its ER luminal location. Conversely, the sensitivity to mutation of the small loop between TMS1 and TMS2 is consistent with its cytosolic localization However, it should be stressed that helices observed in TMS2 and TMS3 are not classical membrane anchoring TM helices, since they contain polar and charged residues. In addition, the TMS2 helix exhibits an amphipathic character suggesting that it could associate with the membrane interface, at least transiently. Based on physicochemical considerations, a transmembrane orientation of this helix is expected to be achieved only upon interaction with another complementary transmembrane segment neutralizing the polar and charged residues located in the hydrophobic core of the membrane. In this context, it is possible that the transmembrane association of TMS2 and TMS3 occurs in the translocon during NS2 biosynthesis. Alternatively, these TMS might be first released into the cytosol where they could interact at the membrane interface and then associate with the membrane to adopt their final transmembrane topology. Interestingly, the length of the connecting loop between TMS2 and TMS3 and the absence of an interaction between these TMS suggest that TMS3 might be an independent entity possibly interacting with distant partners. The fact that chimeric genomes with high assembly competence can be obtained when using a cross-over site right after TMS1 of NS2 indicates that TMS1 is functionally separated from the remainder of the NS2 MBD [14]. Overall, the MBD of NS2 appears to be composed of a series of structural elements with own functional properties, but with the capacity to acquire new functions upon intra- and intermolecular interactions. This structural plasticity is likely essential to ensure the multiple interactions mediated by the NS2 MBD.

### Genetic analysis of NS2 MBD

Almost all helix-swap mutations reduced assembly competence arguing for genotype specific incompatibilities between individual TMS of either NS2 or other viral proteins, such as p7 and E2. The only exception was mutant CT3.h1 affecting helix1 of TMS3 that acts most likely as an adaptable linker between TMS2 and TMS3. This helix is the least conserved sequence of the NS2 MBD suggesting that it mediates interactions with the membrane in a genotype-independent manner.

Selection of assembly-impaired NS2 mutants for titer enhancing mutations compensating the assembly defect to the most part lead to pseudoreversions within NS2. This was the case for all helix-swap mutants and several single aa exchanges. Six out of 9 pseudoreversions within NS2 were found in the loop region connecting TMS1 and 2. This loop resides on the cytosolic side of the ER membrane and by interaction with membrane phospholipids it may stabilize membrane association of NS2 or is involved in intra- or intermolecular protein-protein interactions. Overall, these pseudoreversions most likely restore structural alterations induced by the primary mutation.

Based on our model of the NS2 MBD (Figure 1D), at least some of these mutations could be explained. One example is the Y39A pseudoreversion compensating the assembly defect caused by the G25R mutation suggesting that aa residues 25 and 39, which are located on TMS1 and TMS2, respectively, might be in contact (Figure 10). We assume that the “hole” created in TMS2 by the Y39A substitution is compensated by a bulky aa in the interacting TMS1 counterpart, thus ‘filling up’ the hole in the mutated TMS2. This interaction likely occurs at or close to the membrane interface, where the charge of Arg is well tolerated. Importantly, this assumption is corroborated by the G25R pseudoreversion that was selected with helix-swap mutant JFH1-CT2.h, which has a histidine residue at position 25. We therefore conclude that the beginning of the loop between TMS1 and TMS2 likely interacts with the helix in TMS2. Another example are the pseudoreversions K172R and T21A in NS2 that were selected with helix-swap mutants JFH1-CT3.h2 and JFH1-CT3.h3, respectively, suggesting interactions between TMS1 and TMS3 (Figure 10). While this can be easily explained for position 21, the aa at position 172 is more remote from the membrane surface. Nevertheless, this residue is at the junction between the two subdomains of the NS2 protease domain and thus still suitable to contact TMS3.

We tried to integrate all these informations into our NMR-based structure model of NS2 MBD, but these attempts were confounded by the fact that NS2 is a dimer, which most likely forms higher-order oligomeric complexes. Therefore, we do not know whether a given mutation restores intra- or intermolecular interactions. Nevertheless, the tight correlation between structural integrity of NS2 and its role in assembly is underlined by the fact that titer-enhaning mutations within NS2 have also been found by us and others when using JFH1 wild type or various virus chimeras with low assembly competence [10], [44]–[49].

Apart from pseudoreversions within NS2, we also identified two in p7. Importantly, in case of the helix-swap mutant affecting TMS1, the pseudoreversion in p7 (E3D) enhanced virus production almost back to wild type level. This result argues for an interaction between TMS1 of NS2 and p7 (Figure 10). Unfortunately, this assumption could not be tested directly, because this mutation destroyed the epitope recognized by the p7-specific antibody. However, we have earlier described that for most virus chimeras the best junction for fusion of the genome segments resides after TMS1 of NS2, whereas an intergenotypic fusion right after p7 was severely impaired in assembly [14]. Thus, genotypic compatibility between TMS1 of NS2 and the structural proteins as well as p7 appears to be required for efficient assembly.

A direct interaction between NS2 and envelope glycoproteins might be suggested by the pseudoreversions V341A residing in the N-terminus of the TMS of E2 and mutation I181S in E1 (Figure 10). We note that V341 in E2 and the primary NS2 mutation F77 responsible for the assembly defect are both most likely located in the membrane hydrophobic core, close to the ER membrane interface (Figure 1D for NS2 and Figure 1 in [50] for E2). Moreover, I181S in E1 selected as pseudoreversion with the helix-swap mutant CT3.h2 resides in the center of the TMS of E1 and thus could also directly form a stable in-membrane interaction with NS2 (Figure 10).

W35F and W36L independently adapted via the Q221L pseudoreversion in NS3 that has also been found in two earlier reports [25], [51]. This reversion is highly potent and restores viral infectivity up to ∼38,000-fold. Interestingly, this NS3 mutation also rescues assembly in trans showing that the replication and assembly function of NS3 can be separated genetically. While the mechanism by which Q221L enhances assembly is not known, we note that this residue resides on the helicase NTPase subdomain surface in a basic patch and is well accessible. This positively charged surface area might interact with the membrane surface by electrostatic interactions. In this way the aa residue at position 221 could contact the NS2 MBD at the membrane interface, at least transiently (Figure 10). According to this hypothesis, the replacement of the polar residue (Q) by a large hydrophobic aa (Leu) might reinforce membrane binding.

### Protein interaction and subcellular localization of NS2

Co-immunoprecipitation studies revealed stable interactions of NS2 with NS3, p7 and E2 whereas interaction with NS5A was rather weak. Importantly, none of the tested conditions revealed NS2 interaction with core. These results were well supported by immunofluorescence studies demonstrating a profound and rapid colocalization of NS2 with E2 and NS3 at the ER or an ER-derived membrane compartment prior to accumulation around LDs. Several lines of evidence suggest that NS2 recruits E2 –and thus most likely also E1 that forms a very stable E1/E2 heterodimer [52]– and eventually also p7 to assembly sites in close proximity of LDs. First, we detected a profound colocalization of NS2 and E2 for each time point after infection or transfection; second, in NS2 assembly-defective viruses E2 localized primarily to the ER; third, upon insertion of the corresponding pseudoreversion E2 and NS2 colocalized again to LDs. The NS2-independent LD localization of NS5A and its weak interaction with NS2 is in agreement with previous data showing that NS5A expressed on its own is targeted to LDs, for which the N-terminal amphipathic helix appears to be most critical [33].

No significant colocalization of NS2 and core at LDs was detected. However, a small fraction of core protein presumably residing at the ER colocalized with NS2 both at the early and the late time points after transfection. Although LDs have been described as sites of HCV assembly [31] the weak NS2 – core colocalization is not in contradiction to this observation. In fact, it is speculated that the early steps of HCV assembly (nucleocapsid formation) might take place at LDs whereas the envelopement is thought to occur at the ER or an ER-derived compartment. Since NS2 probably acts at a late step of assembly [27] and might be involved in envelopment of the nucleocapsid, the colocalization of core and NS2 at the ER in close proximity of LDs rather than directly on LDs would support such a model. Moreover, given the complex membrane topology of NS2 this protein most likely can not move onto the surface of LDs that is formed by a membrane monolayer.

### A hypothetical model how NS2 contributes to HCV assembly

The results described in this study together with earlier reports [25], [27] invite speculation how NS2 might contribute to assembly. It is assumed that the early steps (nucleocapsid formation) occur in close proximity of LDs that may serve as assembly platforms [31]. By interaction between core and (RNA-containing) NS5A, capsid formation might be triggered [32]. How the envelope is acquired is not known, but we assume that NS2 plays a central role in this step. Since the TMS of E1 and E2 lack a cytosolic domain that could interact with the core protein, an adaptor protein such as NS2 that in turn efficiently binds p7 and E2 (and the latter forming heterodimers with E1), might be required to ‘deliver’ the envelope proteins to assembly sites in close proximity of LDs. This process could be facilitated by a particular membrane lipid environment supporting recruitment of the NS2 complex as well as the (lipid-binding) nucleocapsid. Alternatively, one or several host cell factors such as CIDE-B, described as an NS2 interactant [53] and required for lipid homeostasis [54], might be recruited by NS2 and aid in assembly. Moreover, NS2 efficiently binds to NS3 arguing that NS2 can form an additional complex with the replicase. How such a complex would contribute to assembly is unclear, but it may ‘tether’ the replicase to the assembly sites thus facilitating core – NS5A interaction. Alternatively, NS2 may form just one higher-order protein complex including in addition to E1/E2 and p7 NS3. This is probably facilitated by the N-terminal MBD that might form ‘clusters’ within the membrane. Finally, it is possible that the strong NS2 - NS3 interaction affects cleavage at the NS2-3 site, in this case contributing to assembly in a rather indirect manner.

In conclusion, our results point to a central role of NS2 in HCV assembly by formation of (a) multiprotein complex(es) with structural and eventually also nonstructural proteins and recruiting them to assembly sites in close proximity of LDs. In this respect, NS2 acts as a central organizer of HCV virion formation.

## Materials and Methods

### Sequence analyses and predictions

Sequence analyses were performed by using Network Protein Sequence Analysis (NPSA) (http://npsa-pbil.ibcp.fr [55]) and European HCV Database (http://euhcvdb.ibcp.fr [56]). Multiple-sequence alignments were performed with CLUSTAL W [57], by using the default options. Protein secondary structures were deduced from a large set of prediction methods available at the NPSA website, including HNNC, SIMPA96, MLRC, SOPM, PHD, and Predator (http://npsa-pbil.ibcp.fr/NPSA and references therein). Octanol hydrophobicity plots were generated with MPEx (http://blanco.biomol.uci.edu/mpex/) by using the scale developed by Wimley and White [58].

### Cell culture

Monolayers of the highly permissive cell lines Huh7-Lunet [59] and Huh7.5 [60] were grown in Dulbecco's modified minimal essential medium (DMEM; Life Technologies, Karlsruhe, Germany) supplemented with 2 mM L-glutamine, nonessential amino acids, 100 U/ml of penicillin, 100 µg/ml of streptomycin, and 10% fetal calf serum. Owing to highest permissiveness for JFH-1, Huh7.5 cells were used for virus production and infection assays whereas Huh7-Lunet cells and derivatives thereof were used for immunofluorescence analyses because of their superior morphology as compared to Huh7.5 cells.

### Plasmids and DNA cloning

NS2-GFP fusion constructs were derived from pFK1-9605Con1 ([7]; HCV Con1 strain). First, a BamHI restriction site was eliminated by introducing a silent mutation replacing the cytidine at nucleotide position 2920 by an adenosine. Sequences encoding NS2 fragments from codon 1–27, or 27–59, or 60–99, or 1–99 or the complete NS2 coding region were fused to EcoRI and BamHI recognition sequences by PCR and amplified fragments were inserted via these two restriction sites into pCMV-KEB-GFP [61], yielding constructs pCMVNS21-27-GFP, pCMVNS227-59-GFP, pCMVNS260-99-GFP, pCMVNS21-99-GFP and pCMVNS2-GFP.

Unless otherwise stated all mutations were introduced into JFH1mut4-6 [10] corresponding to the JFH1 genome [9], but containing three virus titer enhancing mutations that do not affect RNA replication (V2153A, V2440L and V2941M). All nucleotide and aa numbers refer to the JFH1 genome (GenBank accession no. AB047639). Single aa substitutions and helix-swap mutations were introduced by PCR-based site-directed mutagenesis or overlap-PCR, respectively, using standard procedures. In case of the helix-swap mutations the following nucleotide sequences of JFH1 were replaced by the corresponding sequences of Con1: nucleotides 2811 - 2839 in case of pFK-JFH1-CT1.h; nucleotides 2879 - 2911 for pFK-JFH1-CT2.h; nucleotides 2967 - 2986 for pFK-JFH1-CT3.h1; nucleotides 3002 - 3025 in case of pFK-JFH1-CT3.h2; nucleotides 3047 - 3070 with pFK-JFH1-CT3.h3. To generate the JFH1 genome encoding a tagged NS2 protein a sequence encoding the peptide YDAPVSGDYKDDDDKGSG (corresponding to the first 5 aa of NS2, a 2 aa flexible linker (SG) containig a BspEI site, a Flag tag and a flexible GSG linker) was inserted by overlap PCR between nucleotide 2779 and 2780 of the JFH1 genome. A silent G to A mutation at position 2794 was introduced to create a BsrGI restriction site whereas the natural BsrGI site at position 7786 was destroyed by a silent A to T mutation. In addition, a silent A to C nucleotide substitution at position 1741 was introduced to create a *dam* methylation site affecting the BspEI cleavage site at this position. To generate genomes with double tagged NS2, oligonucleotides encoding the Flag-, or hexahistidine- or HA-tag fused to the GSG linker were inserted in-frame into the BspEI site.

### *In vitro* transcription and electroporation of HCV RNAs

The experimental procedures used to generate in vitro transcripts from cloned HCV sequences and transfection of Huh-7 cells by electroporation have been described in detail recently [6]. For trans-complementation assays a mixture of 7.5 µg NS2 mutant and 5 µg helper replicon RNA was used. After electroporation, cells were immediately transferred to complete DMEM and seeded as required for the assay.

### Immunohistochemical staining and virus titration

Virus titers were determined as described elsewhere with slight modifications [13]. In brief, Huh7.5 cells were seeded into 96-well plates and fixed 3 - 4 days after infection. For immunohistochemistry we used an antibody specific for the JFH1 NS3 helicase (2E3, generated in cooperation with H. Tang, Florida State University, USA) at a dilution of 1∶100 or the 9E10 monoclonal antibody specific against NS5A protein in dilution 1∶2,000 (NS5A-9E10; kindly provided by C.M. Rice, New York, USA). Bound antibody was detected with a peroxidase-conjugated secondary antibody specific to murine IgG (Sigma-Aldrich) diluted 1∶200 in PBS. Virus titers (50% tissue culture infective dose per ml; [TCID_50_/ml]) were calculated as described recently [62].

### Selection for pseudorevertants

Huh7.5 cells were electroporated with 10 µg in vitro transcript and culture supernatant harvested 72 h later was concentrated by ultrafiltration using an Amicon Ultra Centrifugal Filter Column (Milipore). Naïve Huh7.5 cells were inoculated with this concentrate and continuously passaged up to 6 times. Thereafter, culture supernatants were passaged 4 times on naïve Huh7.5 cells and virus titers were determined by TCID_50_ or immunofluorescence assay. Details of the adaptation method have been described elsewhere [63].

### Preparation of total RNA, amplification of replicon RNA by RT-PCR and cloning of amplified DNA fragments

HCV RNA present in Huh7.5 cells was amplified and cloned as described previously [10], [63]. In brief, total RNA was isolated from a confluent 10 cm-diameter dish of Huh7.5 cells infected with the adapted virus population by using the Nucleo Spin RNAII Kit (Macherey-Nagel, Düren, Germany) as recommended by the manufacturer. One µg total RNA and 50 pmol of primer A9482 (5′-GGA ACA GTT AGC TAT GGA GTG TAC C-3′) were applied for cDNA synthesis by using the Expand-RT system (Roche, Mannheim, Germany) as recommended by the manufacturer. Two to four microliters of the reaction mixture were used to amplify the 5′ half of the HCV genome with the Expand Long Template PCR kit (Roche) according to the instructions of the manufacturer with primers S59-*Eco*RI (5′-TGT CTT CAC GCA GAA AGC GCC TAG-3′) and A4614 (5′-CTG AGC TGG TAT TAT GGA GAC GTC C-3′). PCR products were directly sequenced (mutants G10S, E45R, P73I and F77A) or inserted into pFK-I_389_Luc-EI/NS3-3′/JFH1-dg after restriction with EcoRI and SpeI. Sequence analysis of two independent plasmid clones was performed with an appropriate set of primers.

### Antisera

The following antisera were used in this study: rabbit polyclonal antibody specific for NS2 (NS2-1519; [6]); rabbit polyclonal antibody specific for the core protein (C-830; [64]); rabbit polyclonal antibody specific for NS3 of JFH1 (NS3-4949; [30]); mouse monoclonal antibody specific for JFH1 NS3 (NS3-2E3; generated in co-operation with H. Tang, Florida State University, USA); rabbit polyclonal antibody specific for JFH1 NS5A (NS5A-52; [6]); mouse monoclonal antibody specific for NS5A (NS5A-9E10, kindly provided by C.M. Rice, New York, USA); rabbit polyclonal anti p7-2716 and anti p7-2717 (kindly provided by M. Harris and S. Griffin, Leeds, UK); rabbit polyclonal antibody specific for J6 E2 [6]; mouse monoclonal antibody specific for E2 protein (AP33, kindly provided by Arvin Patel, Glasgow, U.K.); mouse monoclonal anti-Flag antibody, mouse monoclonal anti-HA antibody and mouse monoclonal anti-β-actin, all from Sigma-Aldrich (Munich, Germany). Monoclonal antibody (mAb) 1D3 against protein disulfide isomerase (PDI) was purchased from StressGen (Victoria, BC, Canada).

### SDS PAGE and Western blot

Western blot analysis was performed as described previously [6]. Samples harvested 48 h after transfection were heated for 20 min at 95°C in sample buffer (125 mM Tris/HCl, 2% (w/v) SDS, 5% (v/v) 2-mercaptoethanol, 10% (v/v) glycerol, 0.001% (w/v) bromophenol blue, pH 6.8) and separated by SDS polyacrylamide gel electrophoresis. Proteins were electro-transferred to a polyvinylidene fluoride (PVDF) membrane (PerkinElmer Life Sciences) for 1 h. Membrane was blocked overnight in PBS supplemented with 0.5% Tween (PBS-T) and 5% dried milk (PBS-M) at 4°C prior to 1 h incubation with primary antibody diluted in 2% milk in PBS-T. Membrane was washed 3 times with PBS-T and incubated for 1 h with horseradish-peroxidase conjugated secondary antibody. Bound antibodies were detected after 3 times washing with the ECL Plus Western Blotting Detection System (GE Healthcare Europe, Freiburg, Germany).

### Co-immunoprecipitation

Huh7.5 cells were mock treated or transfected with HCV RNA, and samples were harvested 72 h later by scraping into IP buffer (0.5% n-dodecyl-β-D-maltoside, 100 mM NaCl, 20 mM Tris pH 7,5). After 60 min incubation on ice, cell debris was removed by 15 min centrifugation at 20,000xg. Samples were incubated with HA-specific antibody beads (Sigma Aldrich) over night at 4°C. After three times washing with IP buffer, samples were eluted into sample buffer and separated by electrophoresis into a 11% Tris-Tricine gel as described elsewhere [65]. Proteins were transferred onto PVDF membrane and HCV proteins were detected by Western blot as described above.

### Immunofluorescence and confocal laser scanning microscopy

U-2 OS human osteosarcoma cells grown on glass coverslips were transfected with GFP fusion constructs, fixed 24 to 48 h post transfection with 2% paraformaldehyde, and mounted in SlowFade (Molecular Probes, Eugene, OR). Immunofluorescence staining was performed as described previously [66]. Bound primary antibody was revealed with Alexa-488-conjugated goat anti-mouse antibody (Molecular Probes). Mounted coverslips were examined using a Leica SP5 AOBS confocal laser scanning microscope. Immunofluorescence detection of HCV proteins in Huh7-Lunet cells was conducted in the analogous way with some modifications. Cells were transfected with HCV RNA and fixed 24, 36, 48 and 72 h post-transfection with 4% paraformaldehyde. Bound primary antibodies were detected with a Alexa-488-conjugated goat anti-rabbit antibody or a Alexa-568-conjugated goat anti-mouse antibody (Molecular Probes). LDs were stained with HCS LipidTOX™ Deep Red neutral lipid stain (Molecular Probes). Coverslips were mounted in Fluoromount-G mounting medium (Electron Microscopy Sciences, Ft. Washington, USA) and examined with a Perkin Elmer spinning disk confocal ERS 6Line microscope. Images were deconvolved with the Huygens Essential 3.5 software using a theoretical point spread function. 3D reconstructed images were created using the Volocity 5.3. software package.

### Peptide synthesis and purification

The NS2[27]-[59] and NS2[60-99] peptides representing aa segments 27–59 and 60–99 of NS2 of the Con1 strain (AC number AJ238799) were synthetized by Clonestar Biotech and purified by RP-HPLC (purity >98%).

### Structure determination by CD and NMR

CD, NMR spectroscopy, NMR-derived constraints and structure calculation, and molecular modeling and structure representation were performed by standard approaches as described in materials and methods S1.

### Accession codes

The atomic coordinates for the NMR structures of peptides NS2[27]-[59] and NS2[60-99] and the NMR restraints in 50% TFE are available in the Research Collaboratory for Structural Bioinformatics (RCSB) Protein Data Bank under accession number 2KWT and 2KWZ respectively. The chemical shifts of all NS2[27]-[59] and NS2[60-99] residues have been deposited in the BioMagResBank (BMRB) under the accession number 16886 and 16892, respectively.

## Supporting Information

Figure S1 Sequence analyses and secondary structures of NS2 transmembrane segments as determined by CD spectroscopy and NMR. (A) NS2 secondary structure prediction of the isolates H77, Con1, and JFH1 (genotypes 1a, 1b and 2a, respectively). Numbers in the top refer to residues of the HCV polyprotein and NS2, respectively. Secondary structure predictions are indicated as helical (h, blue), extended (e, red), turn (t, green), or undetermined (coil [c], yellow). Predictions were made as described in materials and methods. Sec. Cons., secondary structure consensus. (B) Octanol hydrophobicity plot generated with MPEx (http://blanco.biomol.uci.edu/mpex/) using the Wimley and White scale of octanol hydrophobicity [58]. The plot shows the mean values using a window of 19 residues. The consensus segments exhibiting a propensity to partition into the hydrophobic core of the lipid bilayer are indicated by the dash bars. (C) Far-UV circular dichroism (CD) analyses of synthetic peptides NS2[27]-[59] and NS2[60-99] in various membrane mimetic environments. CD spectra were recorded in either 50% 2,2,2-trifluoroethanol (TFE) or 1% L-α-lysophosphatidyl choline (LPC), or the following detergents: 100 mM sodium dodecyl sulfate (SDS), 100 mM n-dodecyl-β-D-maltoside (DM), or 100 mM dodecyl phosphocholine (DPC). (D, E) NMR analysis of the peptides in 50% TFE. A summary of sequential (*i*, *i*+1) and medium-range (*i*, *i*+2 to *i*, *i*+4) NOEs is shown in panel D. Sequential NOEs allowing the assignment of proline residues are indicated in red. Asterisks indicate that the presence of a NOE cross peak was not confirmed, because of overlapping resonances. Intensities of NOEs are indicated by the height of the bars. (E) NMR-derived ^1^Hα chemical shift differences (in parts per million) were calculated by subtraction of the experimental values from the reported random coil conformation values in TFE, respectively. [69]. The dashed lines indicate the standard threshold value of ΔHα for an α-helix (−0.1 ppm).
(1.71 MB TIF)

Figure S2 NS3 is able to rescue assembly-deficient NS2 mutants in trans. (A) To determine wether the NS3 mutation Q221L is able to rescue assembly deficient genomes in trans the substitution was inserted into a JFH1 replicon RNA. This construct had a deletion in NS5A (NS5A_Δ_3) that prevents detection with the monoclonal antibody 9E10 thus allowing the selective detection of the assembly mutant that has an unaltered NS5A [6]. Replicon helper RNA (NS3-3′replicon) was co-transfected with assembly deficient NS2 mutants (JFH1mut4-6 or derivatives thereof carrying mutations affecting W35 or W36) at a ratio of 1: 1.5 into Huh7.5 cells. (B) Supernatants were harvested 48 h after transfection and released infectivity was determined by TCID_50_ assay using the NS5A-specific antibody 9E10. A representative result of two independent experiments with standard deviations is shown. Background of the assays was determined by using JFH1-ΔE1E2 (black line and arrow head). (C) Cell lysates of cells transfected with HCV RNAs specified in the bottom were harvested 48 h after transfection and proteins were analyzed by Western blot. Detected proteins are specified in the left of each panel. Note that NS5A_Δ_3 is expressed only from the subgenomic helper RNA whereas full length NS5A is expressed only from the complete JFH1mut4-6 genome or the NS2 mutants derived thereof. Actin was detected on the same blot and served as loading control. The replicon helper RNA did not affect infectivity titer of wild type virus. However, upon co-transfection of the replicon containing the Q221L mutation, the assembly defect of mutants W35F and W36 was rescued ∼20 or 50-fold, respectively in comparison to titers attained in the absence of this helper RNA. Thus, rescue by NS3-Q221L in trans is possible although infectivity titers are ∼100- or 1000-fold lower as compared to rescue in cis.
(1.18 MB TIF)

Figure S3 Intracellular infectivity correlates with infectivity release in viruses carring pseudoreversions within and outside of NS2. JFH1 mutants carrying single aa substitutions (A) or helix swap mutations (B) with severe impact on virion production were used for insertion of pseudoreversions specified in Table 1. The parental mutant and the corresponding double mutant (containing the pseudoreversion) were transfected into Huh7.5 cells and intra- as well as extracellular infectivity was determined 48 h after transfection by TCID_50_ assay. A representative result of two independent experiments with standard deviations is shown. Background of the assays was determined by using JFH1-ΔE1E2 (black line and arrow head). For nomenclature of the mutants see Table 1.
(0.30 MB TIF)

Figure S4 No gross effect of pseudoreversions within and outside of NS2 on RNA replication or stability of HCV proteins. JFH1 mutants carrying mutations specified in the bottom of each panel with or without additional pseudoreversions were transfected into Huh7.5 cells. After 48 h, NS5A, NS3, core and actin (internal loading control) were analyzed by Western blot.
(2.53 MB TIF)

Figure S5 Changes of NS2 subcellular localization in Huh7 cells after infection. Huh7-LunetCD81H cells [62] were infected with JFHmut4-6HAF-NS2 and 36, 48 and 72 h later, NS2 and LDs were detected by using an HA-specific antibody or BODIPY staining, respectively. For each time point, 180 cells were analyzed. Note that the changes of NS2 subcellular localization in these infected cells very well compare to data obtained by RNA transfection (Figure 8).
(0.08 MB TIF)

Figure S6 Colocalization of NS2 and core in Huh7 cells. Huh7-LunetCD81H cells [62] were transfected with JFH1mut4-6HAF-NS2 and 36 h (A) or 72 h later (B) NS2 colocalization with core was determined by immunofluorescence analysis. NS2 was detected with an HA-specific antibody (red), core with a mono-specific antiserum (green) and LDs were labeled with BODIPY (blue). Note the very limited colocalization of core and NS2 around LDs (best visible in the magnifications shown in the bottom right of panel A and B). However, colocalization was detected in a more reticular compartment next to LDs.
(4.21 MB TIF)

Materials and Methods S1 (0.06 MB DOC)
