# Supplementary figures and images for: Structural and Functional Studies of Nonstructural Protein 2 of the Hepatitis C Virus Reveal Its Key Role as Organizer of Virion Assembly
Source: PLoS Pathog. 2010 Dec 16;6(12):e1001233. doi: 10.1371/journal.ppat.1001233 (PMC3002993; doi:10.1371/journal.ppat.1001233)

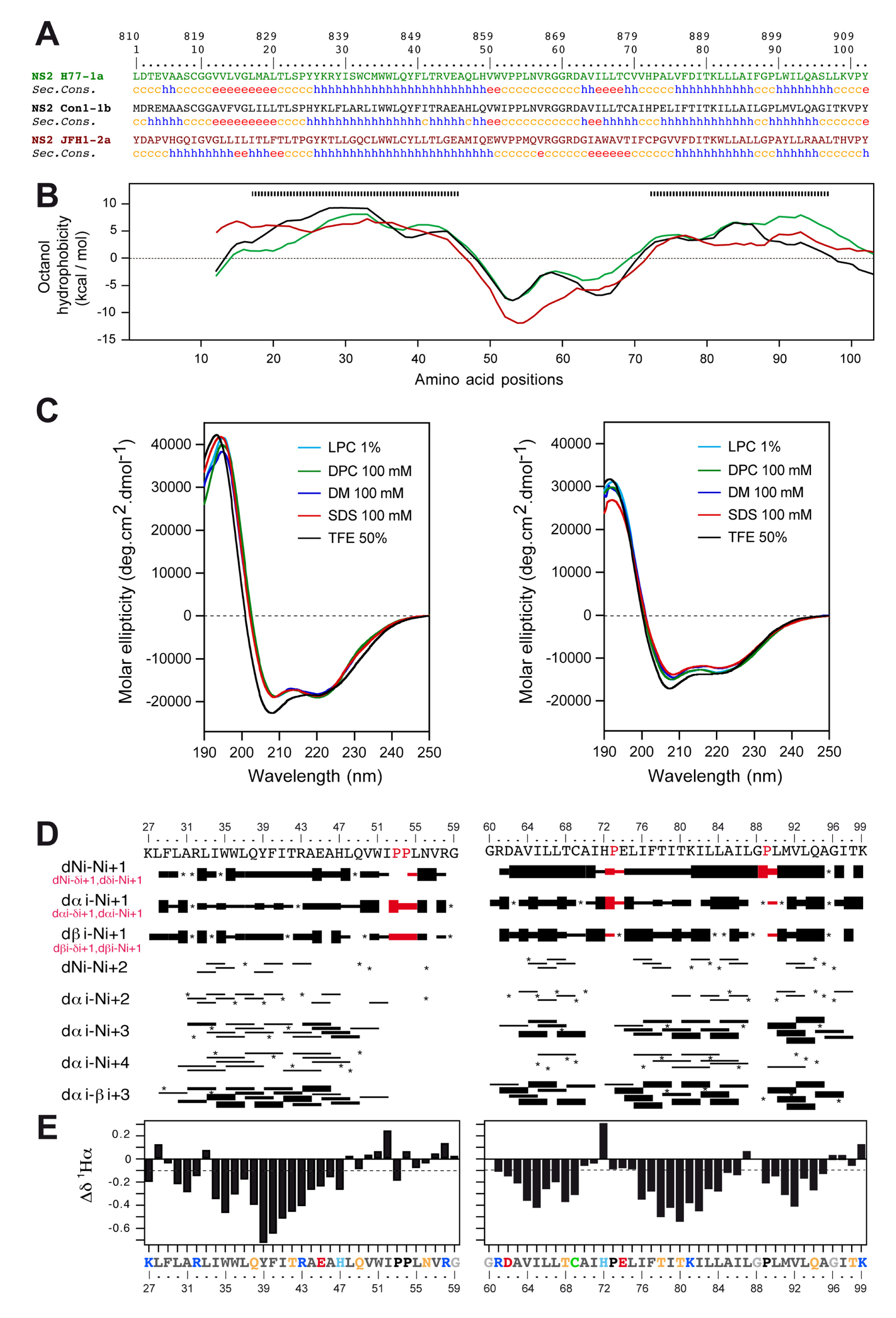

Supplement: Figure S1 — Sequence analyses and secondary structures of NS2 transmembrane segments as determined by CD spectroscopy and NMR. (A) NS2 secondary structure prediction of the isolates H77, Con1, and JFH1 (genotypes 1a, 1b and 2a, respectively). Numbers in the top refer to residues of the HCV polyprotein and NS2, respectively. Secondary structure predictions are indicated as helical (h, blue), extended (e, red), turn (t, green), or undetermined (coil [c], yellow). Predictions were made as described in materials and methods. Sec. Cons., secondary structure consensus. (B) Octanol hydrophobicity plot generated with MPEx (http://blanco.biomol.uci.edu/mpex/) using the Wimley and White scale of octanol hydrophobicity [58]. The plot shows the mean values using a window of 19 residues. The consensus segments exhibiting a propensity to partition into the hydrophobic core of the lipid bilayer are indicated by the dash bars. (C) Far-UV circular dichroism (CD) analyses of synthetic peptides NS2[27]-[59] and NS2[60-99] in various membrane mimetic environments. CD spectra were recorded in either 50% 2,2,2-trifluoroethanol (TFE) or 1% L-α-lysophosphatidyl choline (LPC), or the following detergents: 100 mM sodium dodecyl sulfate (SDS), 100 mM n-dodecyl-β-D-maltoside (DM), or 100 mM dodecyl phosphocholine (DPC). (D, E) NMR analysis of the peptides in 50% TFE. A summary of sequential (i, i+1) and medium-range (i, i+2 to i, i+4) NOEs is shown in panel D. Sequential NOEs allowing the assignment of proline residues are indicated in red. Asterisks indicate that the presence of a NOE cross peak was not confirmed, because of overlapping resonances. Intensities of NOEs are indicated by the height of the bars. (E) NMR-derived 1Hα chemical shift differences (in parts per million) were calculated by subtraction of the experimental values from the reported random coil conformation values in TFE, respectively. [69]. The dashed lines indicate the standard threshold value of ΔHα for an α-helix (−0.1 ppm). (1.71 [file ppat.1001233.s001.tif]

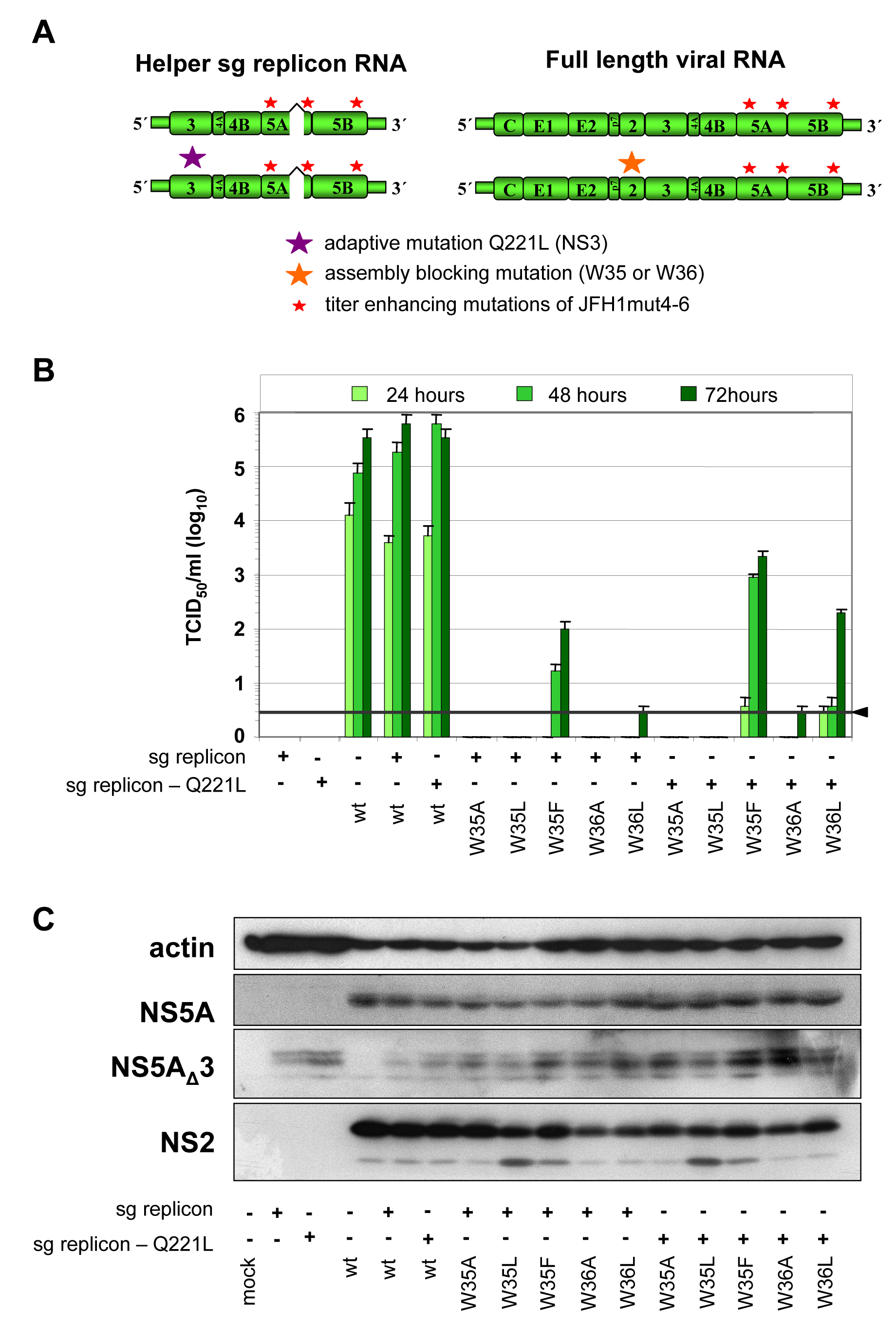

Supplement: Figure S2 — NS3 is able to rescue assembly-deficient NS2 mutants in trans. (A) To determine wether the NS3 mutation Q221L is able to rescue assembly deficient genomes in trans the substitution was inserted into a JFH1 replicon RNA. This construct had a deletion in NS5A (NS5AΔ3) that prevents detection with the monoclonal antibody 9E10 thus allowing the selective detection of the assembly mutant that has an unaltered NS5A [6]. Replicon helper RNA (NS3-3′replicon) was co-transfected with assembly deficient NS2 mutants (JFH1mut4-6 or derivatives thereof carrying mutations affecting W35 or W36) at a ratio of 1: 1.5 into Huh7.5 cells. (B) Supernatants were harvested 48 h after transfection and released infectivity was determined by TCID50 assay using the NS5A-specific antibody 9E10. A representative result of two independent experiments with standard deviations is shown. Background of the assays was determined by using JFH1-ΔE1E2 (black line and arrow head). (C) Cell lysates of cells transfected with HCV RNAs specified in the bottom were harvested 48 h after transfection and proteins were analyzed by Western blot. Detected proteins are specified in the left of each panel. Note that NS5AΔ3 is expressed only from the subgenomic helper RNA whereas full length NS5A is expressed only from the complete JFH1mut4-6 genome or the NS2 mutants derived thereof. Actin was detected on the same blot and served as loading control. The replicon helper RNA did not affect infectivity titer of wild type virus. However, upon co-transfection of the replicon containing the Q221L mutation, the assembly defect of mutants W35F and W36 was rescued ∼20 or 50-fold, respectively in comparison to titers attained in the absence of this helper RNA. Thus, rescue by NS3-Q221L in trans is possible although infectivity titers are ∼100- or 1000-fold lower as compared to rescue in cis. (1.18 MB TIF) [file ppat.1001233.s002.tif]

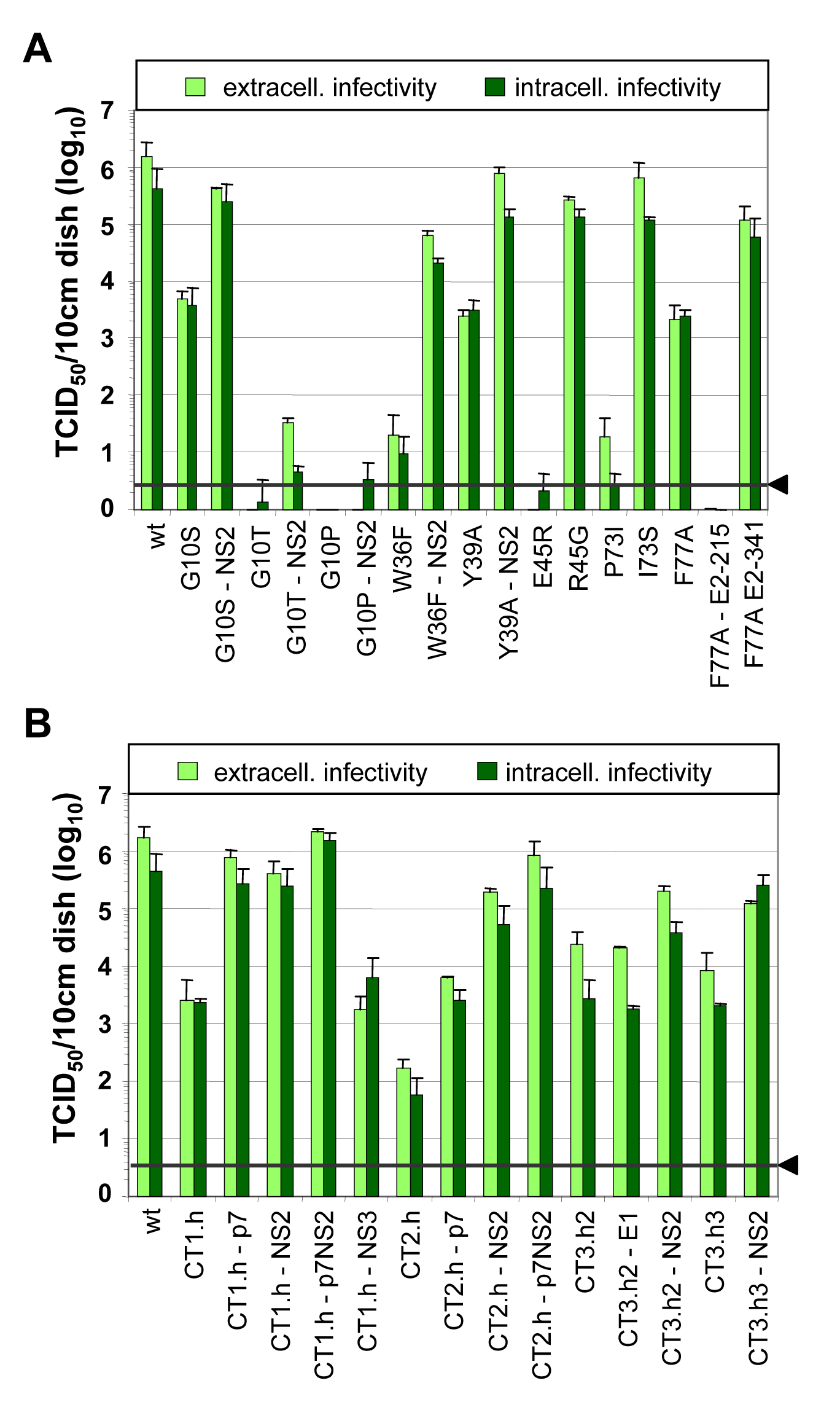

Supplement: Figure S3 — Intracellular infectivity correlates with infectivity release in viruses carring pseudoreversions within and outside of NS2. JFH1 mutants carrying single aa substitutions (A) or helix swap mutations (B) with severe impact on virion production were used for insertion of pseudoreversions specified in Table 1. The parental mutant and the corresponding double mutant (containing the pseudoreversion) were transfected into Huh7.5 cells and intra- as well as extracellular infectivity was determined 48 h after transfection by TCID50 assay. A representative result of two independent experiments with standard deviations is shown. Background of the assays was determined by using JFH1-ΔE1E2 (black line and arrow head). For nomenclature of the mutants see Table 1. (0.30 MB TIF) [file ppat.1001233.s003.tif]

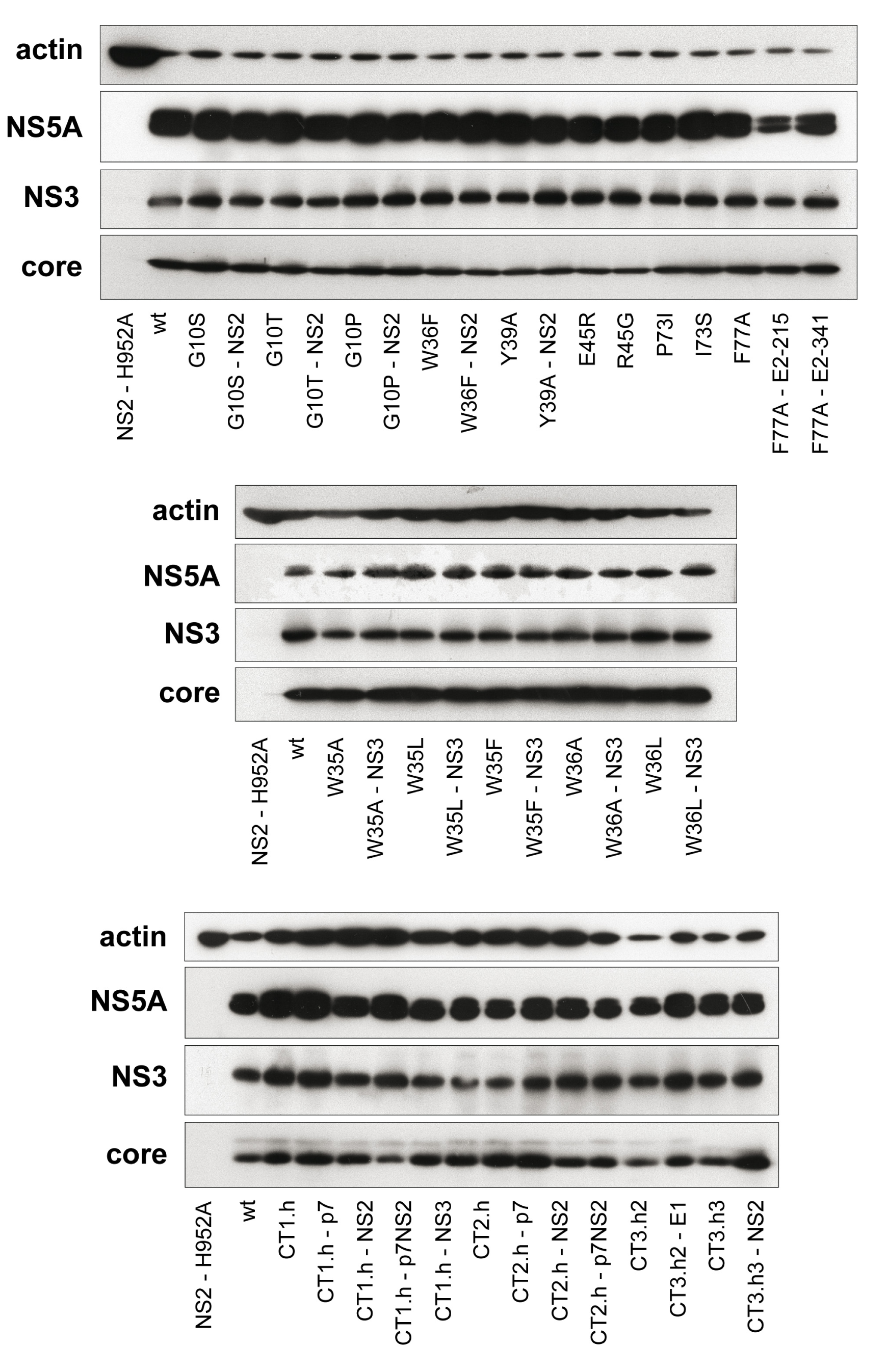

Supplement: Figure S4 — No gross effect of pseudoreversions within and outside of NS2 on RNA replication or stability of HCV proteins. JFH1 mutants carrying mutations specified in the bottom of each panel with or without additional pseudoreversions were transfected into Huh7.5 cells. After 48 h, NS5A, NS3, core and actin (internal loading control) were analyzed by Western blot. (2.53 MB TIF) [file ppat.1001233.s004.tif]

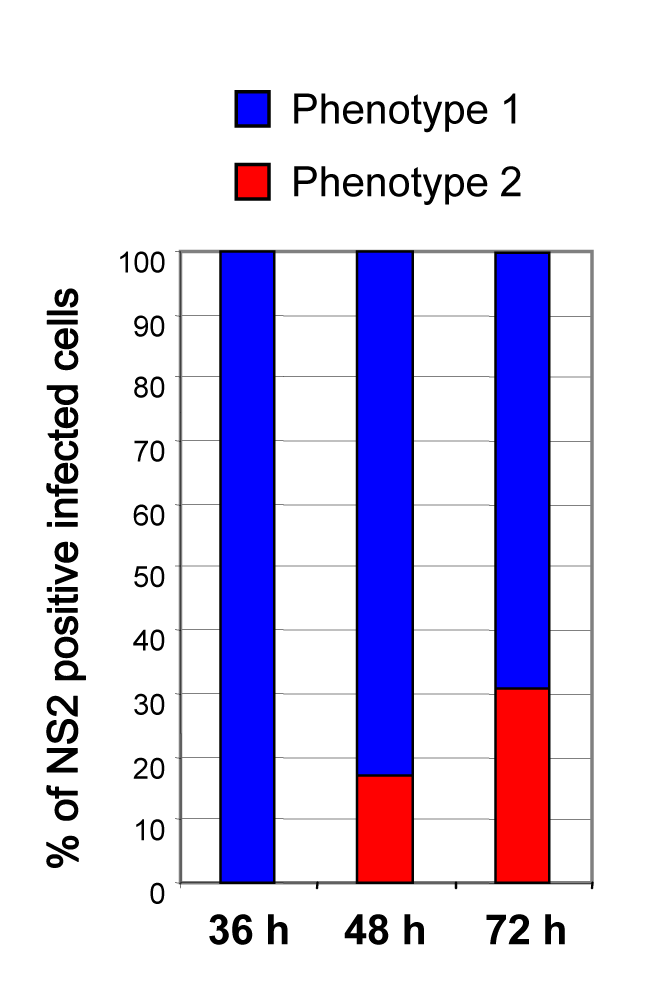

Supplement: Figure S5 — Changes of NS2 subcellular localization in Huh7 cells after infection. Huh7-LunetCD81H cells [62] were infected with JFHmut4-6HAF-NS2 and 36, 48 and 72 h later, NS2 and LDs were detected by using an HA-specific antibody or BODIPY staining, respectively. For each time point, 180 cells were analyzed. Note that the changes of NS2 subcellular localization in these infected cells very well compare to data obtained by RNA transfection (Figure 8). (0.08 MB TIF) [file ppat.1001233.s005.tif]

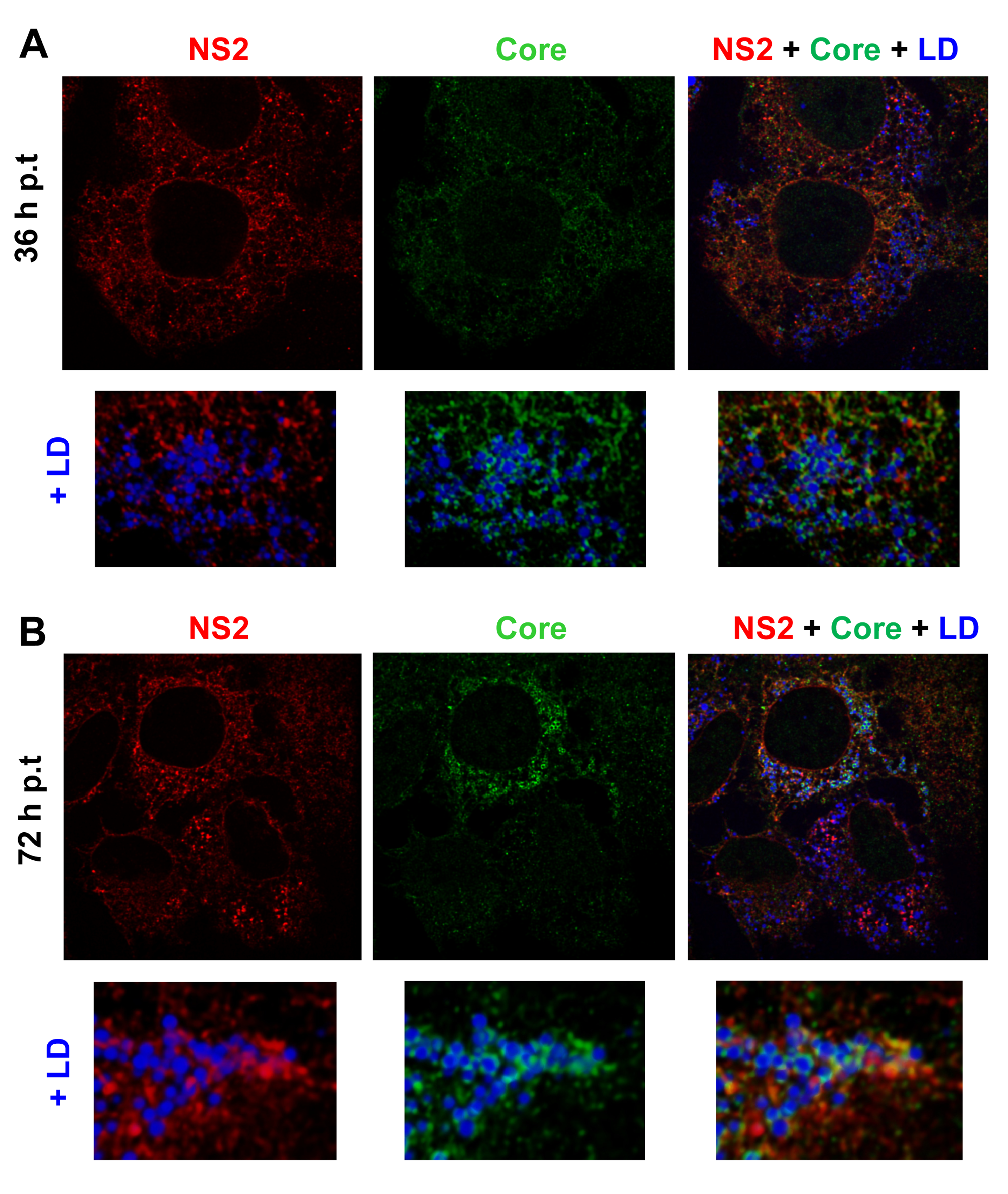

Supplement: Figure S6 — Colocalization of NS2 and core in Huh7 cells. Huh7-LunetCD81H cells [62] were transfected with JFH1mut4-6HAF-NS2 and 36 h (A) or 72 h later (B) NS2 colocalization with core was determined by immunofluorescence analysis. NS2 was detected with an HA-specific antibody (red), core with a mono-specific antiserum (green) and LDs were labeled with BODIPY (blue). Note the very limited colocalization of core and NS2 around LDs (best visible in the magnifications shown in the bottom right of panel A and B). However, colocalization was detected in a more reticular compartment next to LDs. (4.21 MB TIF) [file ppat.1001233.s006.tif]
