# Supplementary material for: Structural and Functional Studies of Nonstructural Protein 2 of the Hepatitis C Virus Reveal Its Key Role as Organizer of Virion Assembly
Source: PLoS Pathog. 2010 Dec 16;6(12):e1001233. doi: 10.1371/journal.ppat.1001233 (PMC3002993; doi:10.1371/journal.ppat.1001233)
Supplement: Materials and Methods S1 — (0.06 MB DOC) [file ppat.1001233.s007.doc]

**Materials and Methods S1**

**DNA cloning and plasmids used for trans-complementation.** Plasmid pFK-JFH1_Q221L_NS3-3´_‎_∆_2328-2435_dg was used. It was derived from pFK-JFH1_NS3-3´_‎dg [1] by introducting a A to T mutation at position 4182. To discriminate in TCID_50_ assays between the virus genome and the helper replicon, a 324-nucleotides long deletion (nucleotide 7322 to 7645 of JFH1) was introduced into domain III of NS5A [2,3]. This deletion removes the epitope that is recognized by the NS5A-specific monoclonal antibody 9E10 [4]. Amplified DNA fragments were validated by automated nucleotide sequence analysis using an ABI 310 sequencer (Applied Biosystems, Darmstadt, Germany). Further details of cloning strategies are available upon request.

**Peptide synthesis and purification.** The NS2[27-59] and NS2[60-99] peptides representing aa segments 27 - 59 and 60 - 99 of NS2 of the Con1 strain (accession number AJ238799) were synthesized by Clonestar Biotech s.r.o. (Brno, Czech Republic) and purified by reverse -phase HPLC on a Nucleosil C8 column (300 Å, 10 µm, 250 mm) using a water/acetonitrile gradient containing 0.1% trifluoroacetic acid. Purity of the peptides (>98%) was checked by RP-HPLC, electrospray mass spectroscopy and NMR spectroscopy.

**Circular Dichroism.** Far UV circular dichroism (CD) spectra were recorded on an Applied Photophysics Chirascan spectrometer calibrated with 1*S*-(+)-10-camphorsulfonic acid. Measurements were carried out at 298 K in a 0.1 cm path length quartz cuvette (Hellma), with peptide concentrations ranging from 6 to 54 µM. Spectra were measured in a 180 nm to 260 nm wavelength range with an increment of 0.2 nm, bandpass of 0.5 nm and integration time of 1 s. Spectra were processed, baseline corrected, smoothed and converted with the Chirascan software. Spectral units were expressed as the mean molar ellipticity per residue by using the peptide concentration determined with absorbance at 280 nm (except for NS2[60-99] where concentration was measured by NMR with tryptophan as an internal standard). The secondary structure content was estimated with various deconvolution methods employing the DICHROWEB server [5].

**NMR spectroscopy.** The purified NS2[27-59] and NS2[60-99] peptides were dissolved either in 100 mM dodecylphosphocholine (DPC)-*d*38 ( 98%) or SDS-*d*25 (98%) or in a mixture of 50% 2,2,2-trifluoroethanol (TFE)-*d*_2_ (>99%) in H_2_O (v/v). 2,2-dimethyl-2-silapentane-5-sulfonate was added to the NMR samples as an internal ^1^H chemical shift reference. All NMR spectra were acquired at 298 K. Multidimensional experiments were performed on Varian Unity-*plus* or Bruker Avance 500 MHz spectrometers using standard homonuclear pulse sequences such as Nuclear Overhauser effect spectroscopy (NOESY; mixing times between 100 and 250 ms) and clean total correlation spectroscopy (TOCSY; isotropic mixing time of 80 ms), as described previously ([6,7] and references therein). VNMR or Topspin softwares were used to process all data and the Sparky software package was used for spectra analyses (T.D. Goddard and D.G. Kneller, University of California, San Francisco). Intraresidue backbone resonances and aliphatic side chains were identified from homonuclear ^1^H TOCSY experiments and confirmed with ^1^H-^13^C heteronuclear single quantum coherence (HSQC) in ^13^C natural abundance. Sequential assignments were determined by correlating intraresidue assignments with interresidues cross peaks observed in two-dimensional ^1^H NOESY. NMR derived ^1^Hα and ^13^Cα chemical shifts are reported relative to the random coil chemical shifts in TFE [8].

**NMR-derived constraints and structure calculation.** NOE intensities used as distance input for structure calculations were obtained from the NOESY spectrum recorded with a 100 ms mixing time and checked for spin diffusion on spectra recorded at lower mixing times (50 ms). NOEs were partitioned into three categories of intensities, which were converted into distances ranging from a common lower limit of 1.8 Å to upper limits of 2.8 Å, 3.9 Å and 5.0 Å, respectively. Protons without stereospecific assignments were treated as pseudoatoms, and the correction factors were added to the upper distance constraints. Neither additional dihedral angle nor hydrogen bond restraints were introduced. The three-dimensional structures were generated from NOE distances by means of a dynamic simulated annealing protocol, using the XPLOR-NIH 2.19 program [9] and standard force field and default parameter sets. Sets of 50 structures were initially calculated to widely sample the conformational space, and the structures of low energy with no distance restraint violations (> 0.5 Å) were retained. Selected structures were compared by pairwise root-mean square deviation (RMSD) over the backbone atom coordinates (N, Cα and C'). Statistical analyses, superimposition of structures, and structural analyses were performed with MOLMOL version 2.6 [10] and the quality of the selected structures was checked with the RCSB PDB validation server.

Reference List

1. Binder M, Quinkert D, Bochkarova O, Klein R, Kezmic N, et al. (2007) Identification of determinants involved in initiation of hepatitis C virus RNA synthesis by using intergenotypic replicase chimeras. J Virol 81: 5270-5283.

2. Appel N, Zayas M, Miller S, Krijnse-Locker J, Schaller T, et al. (2008) Essential role of domain III of nonstructural protein 5A for hepatitis C virus infectious particle assembly. PLoS Pathog 4: e1000035.

3. Jirasko V, Montserret R, Appel N, Janvier A, Eustachi L, et al. (2008) Structural and functional characterization of nonstructural protein 2 for its role in hepatitis C virus assembly. J Biol Chem 283: 28546-28562.

4. Lindenbach BD, Evans MJ, Syder AJ, Wolk B, Tellinghuisen TL, et al. (2005) Complete replication of hepatitis C virus in cell culture. Science 309: 623-626.

5. Whitmore L, Wallace BA (2004) DICHROWEB, an online server for protein secondary structure analyses from circular dichroism spectroscopic data. Nucleic Acids Res 32: W668-W673.

6. Penin F, Geourjon C, Montserret R, Bockmann A, Lesage A, et al. (1997) Three-dimensional structure of the DNA-binding domain of the fructose repressor from Escherichia coli by H-1 and N-15 NMR. J Mol Biol 270: 496-510.

7. Favier A, Brutscher B, Blackledge M, Galinier A, Deutscher J, et al. (2002) Solution structure and dynamics of Crh, the Bacillus subtilis catabolite repression HPr. J Mol Biol 317: 131-144.

8. Merutka G, Dyson HJ, Wright PE (1995) Random Coil H-1 Chemical-Shifts Obtained As A Function of Temperature and Trifluoroethanol Concentration for the Peptide Series Ggxgg. J Biomol Nmr 5: 14-24.

9. Schwieters CD, Kuszewski JJ, Tjandra N, Clore GM (2003) The Xplor-NIH NMR molecular structure determination package. J Magn Reson 160: 65-73.

10. Koradi R, Billeter M, Wuthrich K (1996) MOLMOL: A program for display and analysis of macromolecular structures. J Mol Graphics 14: 51-5, 29-32.
